# Imputation of Gene Expression Data in Blood Cancer and Its Significance in Inferring Biological Pathways

**DOI:** 10.3389/fonc.2019.01442

**Published:** 2020-01-08

**Authors:** Akanksha Farswan, Anubha Gupta, Ritu Gupta, Gurvinder Kaur

**Affiliations:** ^1^SBILab, Department of ECE, Indraprastha Institute of Information Technology-Delhi, New Delhi, India; ^2^Laboratory Oncology Unit, Dr. B.R.A. IRCH, AIIMS, New Delhi, India

**Keywords:** matrix imputation, compressive sensing, machine learning, gene enrichment analysis, AML, CLL, MM, blood cancer

## Abstract

**Purpose:** Gene expression data generated from microarray technology is often analyzed for disease diagnostics and treatment. However, this data suffers with missing values that may lead to inaccurate findings. Since data capture is expensive, time consuming, and is required to be collected from subjects, it is worthwhile to recover missing values instead of re-collecting the data. In this paper, a novel but simple method, namely, DSNN (Doubly Sparse DCT domain with Nuclear Norm minimization) has been proposed for imputing missing values in microarray data. Extensive experiments including pathway enrichment have been carried out on four blood cancer dataset to validate the method as well as to establish the significance of imputation.

**Methods:** A new method, namely, DSNN, was proposed for missing value imputation on gene expression data. Method was validated on four dataset, CLL, AML, MM (Spanish data), and MM (Indian data). All the dataset were downloaded from GEO repository. Missing values were introduced in the original data from 10 to 90% in steps of 10% because method validation requires ground truth. Quantitative results on normalized mean square error (NMSE) between the ground truth and imputed data were computed. To further validate and establish the significance of the proposed imputation method, two experiments were carried out on the data imputed with the proposed method, data imputed with the state-of-art methods, and data with missing values. In the first experiment, classification of normal vs. cancer subjects was carried out. In the second experiment, biological significance of imputation was ascertained by identifying top candidate tumor drivers using the existing state-of-the-art SPARROW algorithm, followed by gene list enrichment analysis on top candidate drivers.

**Results:** Quantitative NMSE results of the DSNN method were compared with three state-of-the-art imputation methods. DSNN method was observed to perform better compared to these other methods both at high as well as low observable data. Experiment-1 demonstrated superior results on classification with imputation compared to that performed on missing data matrix as well as compared to classification on imputed data with existing methods. In experiment-2, cancer affected pathways were discovered with higher significance in the data imputed with the proposed method compared to those discovered with the missing data matrix.

**Conclusion:** Missing value problem in microarray data is a serious problem and can adversely influence downstream analysis. A novel method, namely, DSNN is proposed for missing value imputation. The method is validated quantitatively on the application of classification and biologically by performing pathway enrichment analysis.

## 1. Introduction

### 1.1. Motivation

High dimensional gene expression data helps in determining gene-to-gene interactions in different biological pathways. Molecular techniques such as “Microarrays” are used to measure expression levels of genes. Different machine learning algorithms and statistical methods are applied to gene expression data to extract relevant information for applications such as disease diagnosis and classification of clinical subtypes. These analyses assist in developing effective drugs for specific diseases because treatment procedures differ from disease to disease and case to case. For example, in blood cancer, the drugs are required to be targeted toward the specific type of cancer. Similarly, in personalized therapy, the response of a subject to a particular drug can be captured and its correlation with the mutation profiles of the subject can be examined to design targeted medicine. Thus, the therapy can be customized according to the genetic built of the subject.

A persistent problem associated with microarray dataset is the presence of varying number of missing values in the data that may arise owing to poor slide quality (dusty or scratchy), poor image quality, or insufficient resolution (1). Subsequent downstream analysis on incomplete gene expression matrices may be highly inaccurate. One of the ways of dealing with the problem of missing values is to capture microarray data again but it does not guarantee complete data matrix. Moreover, the entire process is expensive and time consuming. An alternate solution to this problem is to remove the genes containing missing values from the analysis. However, this can result in loss of information and may lead to inaccurate findings on driver genes and/or altered biological pathways. Therefore, it is worthwhile to apply advanced computational methods for the imputation of missing values in microarray data prior to any analysis.

### 1.2. Background

Numerous methods have been developed in the recent times for imputation of gene expression data. These can be broadly categorized into four classes: hybrid methods, local methods, global methods, and knowledge assisted methods (Table 1). Some of the early methods developed to account for the missing values are ZEROimpute, ROWimpute and COLimpute (18). In ZEROimpute, missing values are replaced with zeros. In ROWimpute and COLimpute, missing values are replaced with the averaged values of the observed entries of the corresponding rows or columns. These methods do not take into consideration the correlation present among genes and therefore, do not perform optimally. Gene expression matrix is highly correlated. Therefore, it is important to consider correlation among genes. Several methods exist in literature based on correlation among genes. These are categorized into local and global approaches based on the type of correlation utilized by them. As shown in Table 1, local approaches impute missing values by considering the group of genes that show high correlation with the gene containing missing values. Such methods perform optimally when the data is heterogeneous. *k* nearest-neighbor imputation (KNNimpute) (2) is one of the earliest local approach method to impute missing value. It first estimates *k* nearest group of genes that are similar to the missing target gene, followed by averaging of these genes to impute the missing value of the target gene. SKNNimpute (Sequential KNNimpute) (3) and IKNNimpute (iterative KNNimpute) (4) are variations of KNNimpute. Gaussian mixture clustering imputation (GMCimpute) (5), least square imputation (LSimpute) (6), and variations to LLSimpute, sequential LLSimpute (SLLSimpute) (7), iterative LLSimpute (ILLSImpute) (8), robust least square estimation with principal components (RLSP) (9), Bayesian gene selection BGSregress (10), collateral missing value imputation (CMVE) (11), and auto-regressive least square imputation (ARLS) (12) are all examples of local approaches. On the other hand, SVDimpute (Singular Value Decomposition) (2), Bayesian Principal Component Analysis (BPCA) (13) are the examples of global approach and utilize the global correlation present in the entire gene expression matrix. Hybrid approaches include methods like LinCmb (14), HPM-MI (15), and tri-imputation (16). GOimpute (17), HAimpute (1), and (iMISS) (19) are knowledge-assisted methods that combine the already existing domain knowledge to imputation techniques for imputing missing values in gene expression data, thereby, increasing their imputation accuracy. Gene Ontology based similarity measure has been recently used for missing value imputation in miRNA microarray data (20). A brief review of all the existing methods is shown in Table 1.

**Table 1:** Review of existing methods for missing value imputation in gene expression data.

	**Local approach**	**Global approach**	**Hybrid approach**	**Knowledge assisted approach**
Method	Imputes missing values by first estimating the local correlation among the group of genes that are highly correlated with the gene containing missing values and then using the local correlation to calculate the missing value	Imputes missing values by utilizing the global correlation among the genes in the complete gene expression matrix	Exploits both the global and local correlation among genes to calculate missing values in gene expression data	Imputes missing values by integrating already existing domain knowledge to imputation methods. Information about biological process in the microarray experiment etc. is an example of domain knowledge that can be integrated to the method
Advantages	Perform optimally when the data is heterogeneous i.e., genes exhibit dominant local similarity structure	Perform optimally when the data has high global covariance in expression matrix	Perform optimally regardless of the type of covariance present in the gene expression data	Improves accuracy of missing value imputation and perform optimally in presence of noisy data
Limitations	Perform poorly when data lacks local similarity structure	Fail to perform well when the data is heterogeneous	Perform sub optimally when data is noisy and has high missing rates	Perform sub optimally when data has high missing rates
Examples	*K* nearest-neighbor imputation (KNNimpute) (2) and its variations-SKNNimpute (Sequential KNN) (3), IKNNimpute (iterative KNNimpute) (4) Gaussian mixture clustering imputation (GMCimpute) (5) Least square imputation (LSimpute) (6) and its variations- Local least square imputation (LLSimpute) (3), Sequential LLSimpute (SLLSimpute) (7), iterative LLSimpute (ILLSimpute) (8) and robust least square estimation with principal components (RLSP) (9) Bayesian gene selection BGSregress (10), Collateral missing value imputation (CMVE) (11), Auto-regressive least square imputation (ARLS) (12)	Bayesian Principal Component Analysis (BPCA) (13) SVDimpute (Singular Value Decomposition) (2) first estimates principal components of gene expression matrix by calculating Singular value decomposition of the gene matrix and it then selects the most significant components. These selected components are further used to approximate missing values in the gene expression data	LinCmb (14) uses both global and local correlation information in the data. It estimates missing values using five different imputation algorithms, row average, KNNimpute, GMCimpute, SVDimpute and BPCA. It then takes a convex combination of the results obtained from each of the methods to compute final result HPM-MI (Hybrid Prediction Model with Missing value Imputation) (15) is a hybrid approach that uses both k-means clustering and Multilayer perceptron. It uses eleven different missing value imputation techniques to compute missing values and then selects the best clusters using k-means to compute final result Tri-imputation (16) employs three base imputation algorithms to impute the genes with missing values	GOimpute (17) uses the prior information about the functional similarities in term of GO for missing value imputation HAimpute (Imputation using Histone Acetylation information) (1) combines histone acetylation information as domain knowledge with imputation methods such as KNNimpute and LLSimpute. Accuracy of missing value imputation improves considerably after utilizing domain knowledge

Most of the methods perform missing value imputation in gene expression data at comparatively higher observability, say, when 70% or more data is available (that is equivalent to 30% or less data is missing). Recent developments have made it possible to predict expression data values when the observed data is as low as 10%. Gene expression data is a highly correlated data because of the high level of interdependence between the genes. This interdependence is due to functional relationship between the genes as the group of genes interact together in any biological process. Therefore, it is evident that gene expression matrix is very similar to a low rank matrix that can be embedded into a lower dimensional subspace. Hence, imputation of missing values in data matrix has been projected as the matrix completion problem.

Matrix completion is a popular and challenging area of research in various domains. Many matrix completion methods exist in the literature and out of these methods, LMaFit (Low Rank Matrix Fitting) (21), LogDet (Logarithm determinant) (22), and Robust PCA (RPCA) (23) are three different state-of-the-art matrix completion methods. LMaFit is based on matrix factorization, while LogDet implements nuclear norm minimization. RPCA performs feature reduction and is quite robust to outliers. However, these methods have some limitations. LogDet becomes computationally expensive as the size of the matrix increases. LMaFit and RPCA-GD provide good performance, but their parameters need to be tuned properly for better recovery of missing values. Recently Kapur et al. (24) has used low rank constrained matrix completion method for imputing missing values in genomics.

In this paper, a novel 2-stage method, DSNN (Doubly Sparse DCT domain with Nuclear Norm minimization), has been proposed for predicting missing values in gene expression data using Compressive Sensing (CS) based formulation. In the first stage, missing values were recovered in gene expression data by formulating it as the CS-based reconstruction with double sparsity in the Discrete Cosine Transform (DCT). It has been shown in Gupta et al. (25) that DCT acts as approximate Karhunen-Loève type transform for a large class of signals, particularly, for slowly varying signals. Inspired by this, researchers have used DCT-based CS recovery to impute gene expression data in recent TV-DCT method (26) and CT-NNBI method (27), although only column sparsity was utilized in both these methods, while DSNN utilizes double sparsity.

Matrix obtained in first stage is considered a noisy version of the original matrix. Therefore, in Stage-2, denoising of the matrix recovered from Stage-1 is done by utilizing nuclear norm minimization. It exploits the low rank property of the data matrix. Missing value imputation was performed on four blood cancer dataset at different observability of data (10–90%) using NMSE as evaluation metric. Significance of imputation was validated by two experiments. In the first experiment, classification of normal vs. cancer subjects was carried out. In the second experiment, biological significance of imputation was ascertained by first identifying top 500 genes using SPARROW algorithm (28), followed by KEGG and GO analysis on these top 500 genes. SPARROW (SPARse selected expRessiOn regulators identified With penalized regression) algorithm finds candidate tumor drivers from the “selected expression regulators” (SERs). It defines SERs as the genes that drive dysregulated transcription leading to carcinogenesis. This algorithm regresses the gene expression values on the candidate SERs and provides a rank to each SERs based on the genes expression values of the samples. The method has been described briefly in section 3. Once the ranking was done by SPARROW, top 500 ranked genes from the list were further studied by KEGG (29–31) and GO pathway (32, 33) analysis using a web based application, Enrichr, developed and maintained by Chen et al. (34) and Kuleshov et al. (35).

## 2. Materials and Methods

### 2.1. Dataset Description

Four publicly available microarray gene expression dataset of different cancer types and different population have been used. Dataset-1 is Chronic lymphocytic leukemia (CLL) cancer dataset (GSE50006) submitted by Dana-Farber Cancer Institute, USA. CLL dataset contains expression values of 220 subjects across 54675 probe-ids and consists of two classes depending on whether the subject has CLL or not. There are 188 tumor samples and rest 32 are normal samples. Dataset-2 is Acute myeloid leukemia (AML) cancer dataset (GSE9476) (36) from Fred Hutchinson Cancer Research Centre, USA. It contains gene expression values of 64 subjects across 22283 probe-ids. Two classes are present in the data. Label “1” corresponds to person suffering from AML and label “2” corresponds to healthy subject. There are 26 tumor subjects and 38 healthy subjects. Dataset-3 is Multiple Myeloma (MM) cancer dataset (GSE47552) (37) from Centro de Investigacion del Cancer de Salamanca, Spain. It contains gene expression data of 99 subjects across 33297 probe-ids. It has data from 20 subjects with MGUS, 33 with high-risk SMM, 41 with MM, and rest 5 were healthy subjects. Dataset-4 is Multiple Myeloma (MM) cancer dataset (GSE125361) belonging to Indian population. It contains gene expression data of 48 MM subjects across 58341 probe-ids.

Data was pre-processed to convert probe-ids to gene symbols because gene vs. sample information is required for SPARROW analysis. It was observed that several probe-ids showed same gene names. To overcome this problem, gene expression levels of the probe-ids corresponding to the same gene name were averaged and gene vs. sample matrix was created. After pre-processing, CLL dataset had 220 samples with expression values of 23,348 genes. AML dataset had 64 samples with expression values of 13,650 genes. MM-Spanish dataset had 99 samples with expression values of 23,307 genes. MM-Indian dataset had 48 samples with gene expression values of 33,973 genes. Since the range of gene expression values was very high (of the order of 10^6^) for the CLL dataset, data was *log* transformed to reduce its dynamic range and to ensure that the smaller values were not shadowed by the higher values during the missing data recovery method.

(1) $${\text{X}}_{\text{log-transformed}}(i,j)=log_{10}({\text{X}}_{\text{original}}(i,j)+1)$$

Matrix imputation was carried out on the sample vs. gene matrices. After matrix imputation, only tumor samples of both the dataset were used for SPARROW analysis.

### 2.2. Method

**Figure 1:**
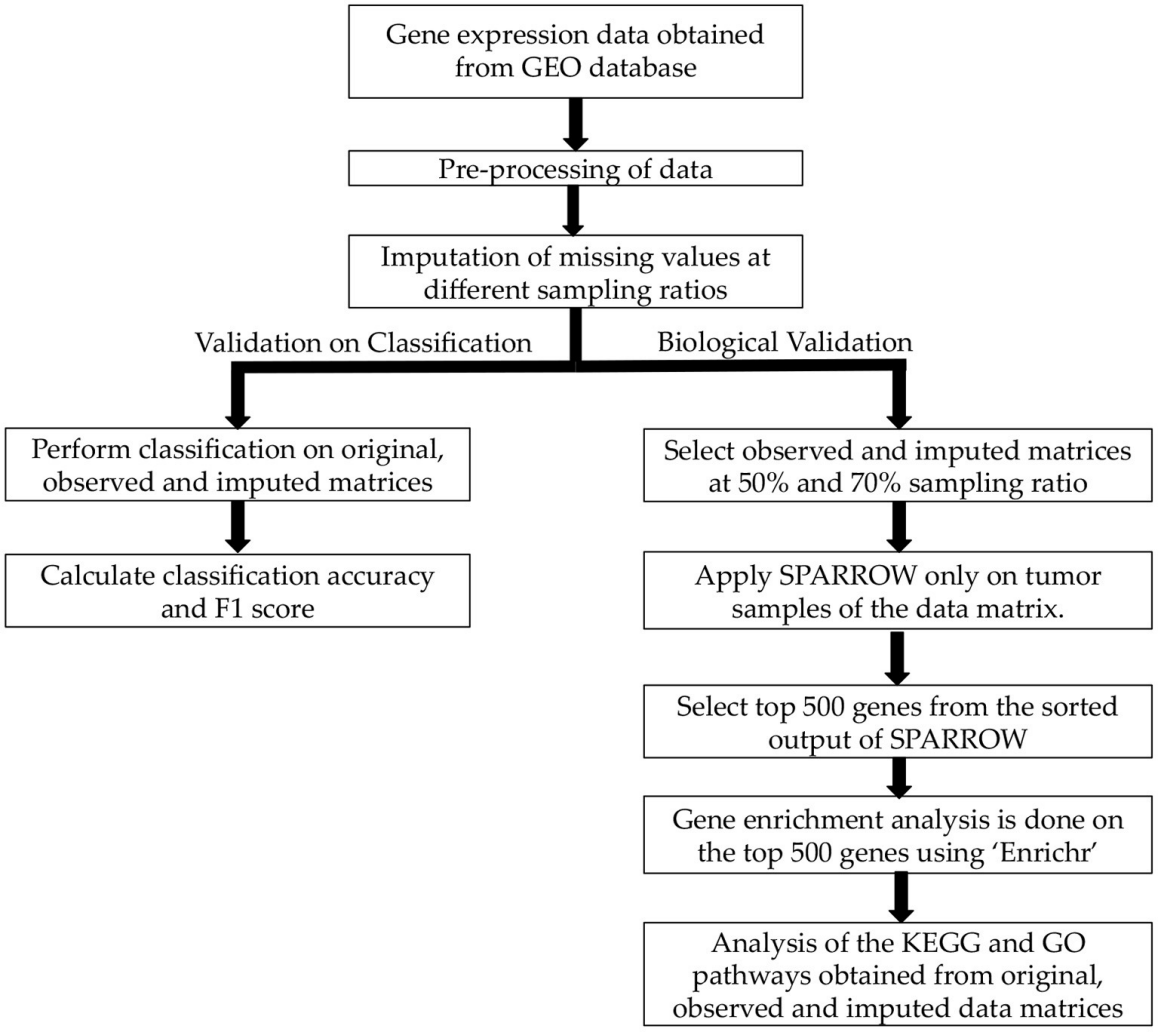
Workflow of the proposed analysis.

Workflow pipeline of the proposed analysis is shown in Figure 1. First of all, pre-processing of raw data was done as described in the previous section. Next, missing value imputation was carried out on four blood cancer dataset at different observability of data using Normalized Mean Square error (NMSE) as evaluation metric. Significance of imputation was validated by two experiments. In the first experiment, classification of normal vs. cancer subjects was carried out. In the second experiment, biological significance of imputation was ascertained using SPARROW algorithm (28) followed by KEGG and GO analysis on the top 500 genes identified by SPARROW.

#### 2.2.1. Proposed DSNN Method of Matrix Imputation

The proposed “Doubly Sparse DCT domain with Nuclear Norm minimization” (DSNN) method consists of two stages. Stage-1 imputes missing values using a CS-based framework and DCT-based sparsity, while Stage-2 removes noise from the matrix obtained from Stage-1 by using a simple denoising framework.

##### 2.2.1.1. Stage-1: Compressive sensing based matrix completion

**Figure 2:**
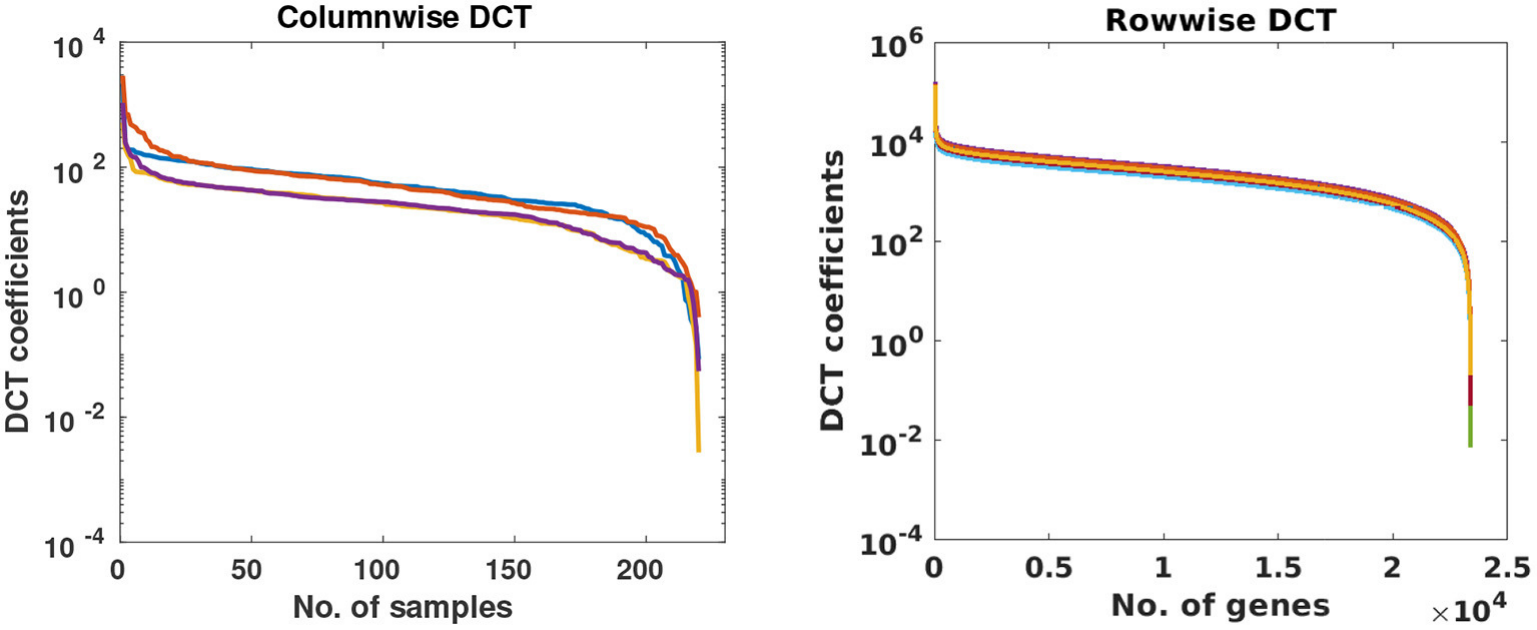
Each curve represents DCT coefficients of a few randomly chosen columns and rows of gene expression matrices of CLL dataset.

In this stage, missing value problem was projected as compressive sensing based reconstruction problem. To understand it better, consider an incomplete matrix **Y** of size *r* × *s*, where *r* represents the number of subjects and *s* denotes the number of genes. Since the expression value of any gene will not vary much across subjects, data within a column would be sparse in some transform domain. Similarly, for a sample, gene expression levels of the gene will also be sparse in some transform domain. Columns and rows of the gene expression matrix were studied in the DCT domain and were observed to be highly sparse as shown in Figure 2. Based on this observation, Discrete Cosine Transform was chosen as the sparsifying transform in DSNN method because DCT acts as a KL-type basis for slow-varying signals (25) and data is sparse in the DCT domain.

Thus, the missing data recovery problem was formulated in a compressive sensing framework, where the sensing matrix Φ was of size *r* × *s* and had “0” entries for missing values in data matrix **Y**, while rest of the entries were “1.” Corresponding to each observed entry (that is not missing) of the *i*th column, there is a row in Φ_*i*_ with an entry “1” for the corresponding position and zeros in the rest of the positions. For example, assume **x**_missing_=${\left[\begin{array}{cccccc} x_{1} & . & x_{3} & . & . & x_{6} \end{array}\right]}^{T}$ is the observed vector where only *x*_1_, *x*_3_, and *x*_6_ are available and, *x*_2_, *x*_4_, and *x*_5_ are missing (denoted as “.” in the vector). Then, the vector **x**_missing_ can be re-written as **y**:

(2) y=Φx

(3) $$\text{y}=\left[\begin{array}{c} x_{1}\\ x_{3}\\ x_{6} \end{array}\right]=\left[\begin{array}{cccccc} 1 & 0 & 0 & 0 & 0 & 0\\ 0 & 0 & 1 & 0 & 0 & 0\\ 0 & 0 & 0 & 0 & 0 & 1 \end{array}\right]\left[\begin{array}{c} x_{1}\\ x_{2}\\ x_{3}\\ x_{4}\\ x_{5}\\ x_{6} \end{array}\right],$$

where the sensing matrix is written as $\Phi =\left[\begin{array}{cccccc} 1 & 0 & 0 & 0 & 0 & 0\\ 0 & 0 & 1 & 0 & 0 & 0\\ 0 & 0 & 0 & 0 & 0 & 1 \end{array}\right]$ and **x** is the desired vector to be recovered. This is the standard formulation in compressive sensing literature, where it is assumed that only few values of data are sensed. In the above example, these values are *x*_2_, *x*_4_, and *x*_5_. Thus, we have recast the problem of missing values in vector **x**_missing_ as the compressively sensed vector **y**. Now, the task is to recover full data **x** from compressively sensed data **y** that will lead to missing value recovery.

Gene expression data was interpreted as a matrix with few observed samples, where the goal was to reconstruct the original matrix from the observed entries using DCT-based sparsity of gene expression data.

The following optimization problem was solved to recover the missing values in **Y**

(4) $$\underset{\tilde{\text{X}}}{min}({\left\|\text{Y}-\Phi \tilde{\text{X}}\right\|}_{2}^{2}+\lambda_{1}{\left\|{\text{D}}_{c}\tilde{\text{X}}{\text{D}}_{r}^{T}\right\|}_{1}),$$

where **D**_*c*_ is columnwise DCT matrix applied on columns of the $\tilde{\text{X}}$ and **D**_*r*_ is the rowwise DCT matrix applied on rows of the $\tilde{\text{X}}$. $\tilde{\text{X}}$ is the matrix to be recovered. The above formulation is also known as analysis-prior and presence of DCT matrices in the formulation makes it non-separable. Using the orthogonal property of DCT transform, analysis prior was transformed to synthesis-prior formulation as

(5) $$\underset{\text{Z}}{min}({\left\|\text{Y}-\Phi {\text{D}}_{c}^{T}\text{Z}{\text{D}}_{r}\right\|}_{2}^{2}+\lambda_{1}{\left\|\text{Z}\right\|}_{1}),$$

where ${\text{D}}_{c}\tilde{\text{X}}{\text{D}}_{r}^{T}=\text{Z}$. The above optimization problem was solved using the function handle and “SPGL1” solver (38, 39), where the regularization parameter λ_1_ was chosen automatically by the “SPGL1” solver.

##### 2.2.1.2. Stage-2: denoising framework

It was assumed that the recovered $\tilde{\text{X}}$ from Stage-1 is the noisy version of the original matrix **X** and hence, the recovered matrix was denoised in Stage-2. Before denoising, $\tilde{\text{X}}$ was re-organized into ${\tilde{\text{X}}}_{\text{rec}}$ as

(6) $${\tilde{\text{X}}}_{\text{rec}}(j,i)=\{\begin{array}{ll} 0, & \text{if}(|\tilde{\text{x}}(j,i)-\text{mean}({\text{y}}_{\text{i}})|\geq \lambda_{\text{2}}\text{std}({\text{y}}_{\text{i}})\\ \tilde{\text{x}}(j,i), & \text{otherwise} \end{array}$$

where *j* ranges from 1 to *m* (number of rows/ subjects), |.| denotes the absolute value and, mean(**y**_*i*_) and std(**y**_*i*_) denote the mean and the standard deviation of the *i*th column of the initial observed (but incomplete) matrix **Y**. Parameter λ_2_ was determined empirically and was set to value 0.2 for experiments on CLL dataset, MM-Spanish dataset, and MM-Indian dataset. It was set to 0.1 for experiments on AML dataset. Denoising was formulated in the Split-Bregman type optimization as

(7) $$\underset{\text{W}}{min}({\left\|\text{W}\right\|}_{*}+\lambda_{3}{\left\|\text{W}-\hat{\text{X}}-\text{B}\right\|}_{F}^{2})\;s.t.\;\hat{\text{X}}=\text{W},$$

where $\hat{\text{X}}$ was initialized as:

(8) $$\hat{\text{X}}={\tilde{\text{X}}}_{\text{rec}}+{\tilde{\text{X}}}_{\text{inv-rec}}◦rand(m,n),$$

where “°” represents the Hadamard product of two matrices with the elements of ${\tilde{\text{X}}}_{\text{inv-rec}}$ defined as

(9) $${\tilde{\text{X}}}_{\text{inv-rec}}(j,i)=\{\begin{array}{ll} 1, & \text{if }{\tilde{\text{X}}}_{\text{rec}}(j,i)=0,\\ 0, & \text{otherwise}. \end{array}$$

Equation (5) was solved in Split Bregman type iterations as

(10) $${\text{W}}^{k+1}=SVT_{\lambda_{3}}({\hat{\text{X}}}^{k}+{\text{B}}^{k}),$$

(11) $${\text{B}}^{k+1}={\hat{\text{X}}}^{k}+{\text{B}}^{k}-{\text{W}}^{k},$$

(12) $${\hat{\text{X}}}^{k+1}={\tilde{\text{X}}}_{\text{rec}}+{\tilde{\text{X}}}_{\text{obs}}◦{\text{W}}^{k+1},$$

where “SVT” denotes the soft singular value thresholding method (40) and ${\tilde{\text{X}}}_{obs}$ is the observed incomplete matrix. Optimal value of parameter λ_3_ was determined using grid search and was set to 100 in all experiments. The complete algorithm for the proposed DSNN method is presented below.

**Algorithm 1:** Proposed DSNN Method

**1 Stage 1 - Compressive sensing based matrix recovery** **Input**: **Y** (Given incomplete matrix), ϕ, Discrete Cosine Transform matrices **D**_*r*_, **D**_*c*_
**2** Obtain **Z** by solving $\underset{\text{Z}}{min}\left(||\text{Y}-\Phi {\text{D}}_{c}^{T}\text{Z}{\text{D}}_{r}|{|}_{2}^{2}+\lambda_{1}||\text{Z}|{|}_{1}\right)$ using ‘spgl' solver
**3** $\tilde{\text{X}}={\text{D}}_{c}^{T}\text{Z}{\text{D}}_{r}$
**Output**: $\tilde{\text{X}}$
**4** **Stage 2: Nuclear-norm based denoising** **Input**: $\tilde{\text{X}}$ (Recovered Matrix from Stage-1 considered as the noisy matrix)
**5** ${\tilde{X}}_{\text{rec}}(j,i)=\{\begin{array}{ll} 0, & \text{if}(\text{|}\tilde{x}(\text{j},\text{i})\text{-mean}(y_{\text{i}})\text{|}\geq \lambda_{\text{2}}\text{std}(y_{\text{i}})\\ \tilde{x}(j,i), & \text{otherwise}, \end{array}$
**6** $\hat{\text{X}}={\tilde{\text{X}}}_{\text{rec}}+{\tilde{\text{X}}}_{\text{inv-rec}}◦rand\left(m,n\right)$
**7** *while* converge:
**8** ${\text{W}}^{k+1}=SVT_{\lambda_{3}}\left({\hat{\text{X}}}^{k}+{\text{B}}^{k}\right)$
**8** ${\text{B}}^{k+1}={\hat{\text{X}}}^{k}+{\text{B}}^{k}-{\text{W}}^{k}$
**9** ${\hat{\text{X}}}^{k+1}={\tilde{\text{X}}}_{\text{rec}}+{\tilde{\text{X}}}_{\text{obs}}◦{\text{W}}^{k+1}$
**10** *end* *while* **Output**: $\hat{\text{X}}$ (Recovered Matrix)

## 3. Results

### 3.1. Evaluation

For assessing the performance of the proposed DSNN method, some data were dropped randomly to create incomplete matrices with available data ranging from 10 to 90%. Next, incomplete matrices were imputed using the DSNN method. Results were simultaneously generated using three state-of-the-art matrix completion methods for comparative analysis. Normalized mean squared error (NMSE) was used as the evaluation metric and was calculated between the original and the recovered matrix. NMSE is defined as:

(13) $$\text{NMSE}=\frac{{\left\|\text{X}(original)-\hat{\text{X}}(recovered)\right\|}_{F}^{2}}{{\left\|\text{X}(original)\right\|}_{F}^{2}}.$$

**Figure 3:**
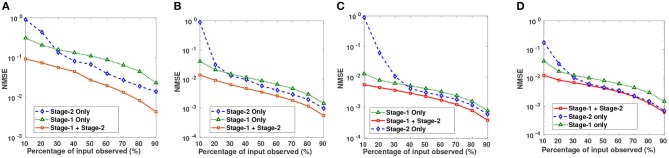
Semi-log plots show NMSE after imputation on **(A)** CLL, **(B)** AML, **(C)** MM-Spanish, and **(D)** MM-Indian dataset using Stage-1 only, Stage-2 only, and Proposed DSNN method (Stage-1 + Stage-2).

**Figure 4:**
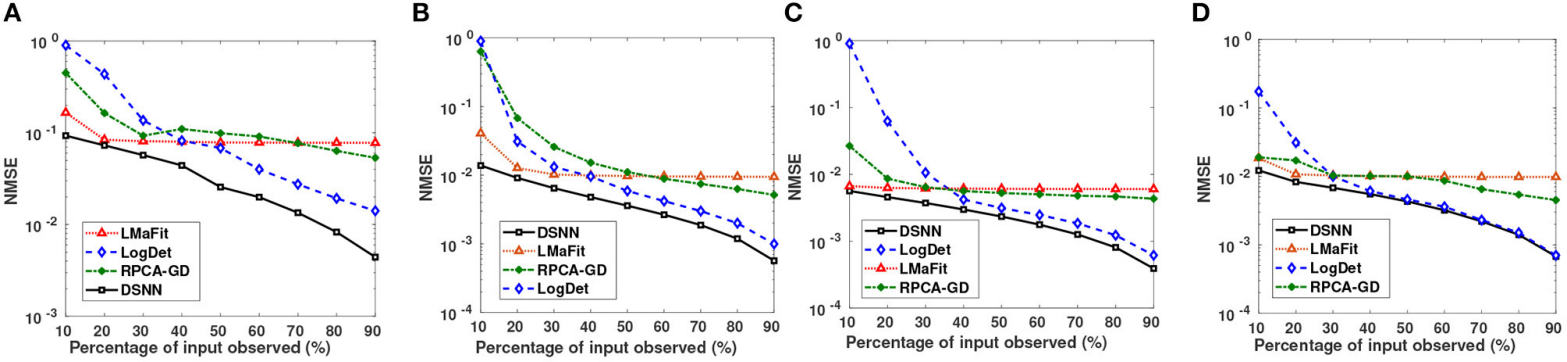
Small Semi-log plots showing comparison of the proposed DSNN method with the three state-of-the-art methods in terms of NMSE for **(A)** CLL, **(B)** AML, **(C)** MM-Spanish, and **(D)** MM-Indian dataset.

Semi-log plots of NMSE at different stages are shown in Figure 3. Stage-1 results were obtained when missing values in data matrix were imputed using compressive sensing based matrix completion, where double sparsity in DCT domain was exploited. Stage-2 results were obtained when only nuclear norm minimization was used for matrix imputation. DSNN method combined both these stages. Results clearly indicated that the performance of imputation has improved with the two successive stages of DSNN. DSNN method also worked better than the existing methods even at high missing rates of 10% as shown in Figure 4. NMSE reported in the figures is averaged over 30 iterations. For CLL dataset, highest NMSE reported was 0.09 at 10% observed data and lowest NMSE was 0.004 at 90% observed data. For AML dataset, highest NMSE was 0.013 at 10% observed data and lowest NMSE was 0.00056 at 90% observed data. For MM-Spanish dataset, highest NMSE reported was 0.005 at 10% observed data and lowest NMSE was 0.00039 at 90% observed data. For MM-Indian dataset, highest NMSE was 0.0122 at 10% observed data and lowest was 6.25E-04 at 90% observed data.

### 3.2. Validation

In order to determine the significance of the DSNN method, two separate experiments were carried out on the original data, incomplete data, and imputed data matrices. In experiment-1, classification of normal vs. cancer subjects was carried out. In experiment-2, biological significance of imputation was ascertained by first identifying top candidate tumor drivers from SPARROW algorithm, followed by gene enrichment analysis on the top-ranked genes using web based application Enrichr.

#### 3.2.1. Experiment 1: Classification

**Figure 5:**
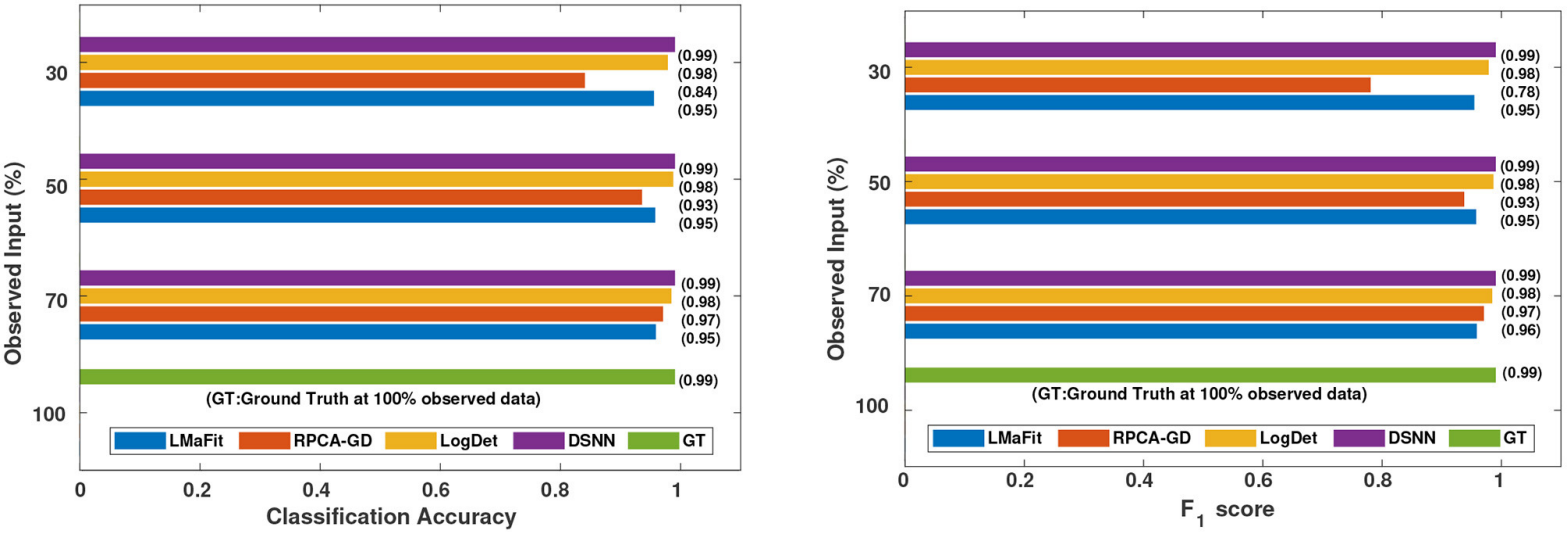
Comparison of different methods in terms of classification accuracy and *F*_1_ score at varying sampling ratios on CLL dataset.

**Figure 6:**
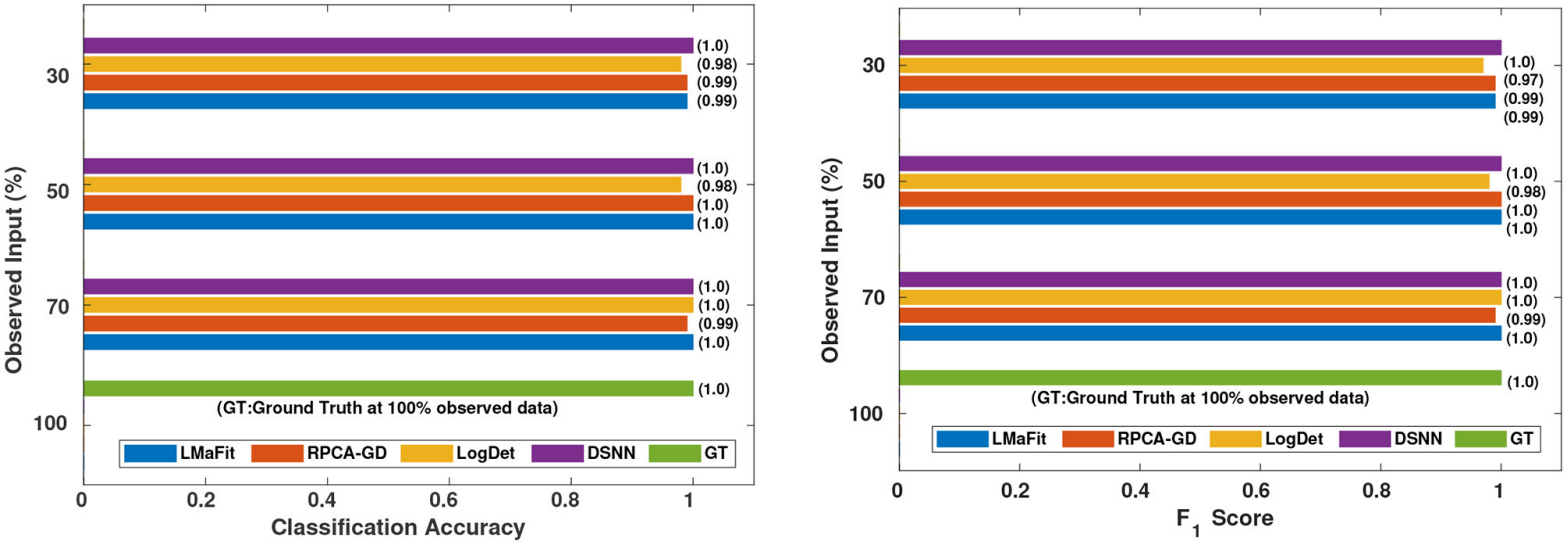
Comparison of different methods in terms of classification accuracy and *F*_1_ score at varying sampling ratios on AML dataset.

Simulation results on missing value recovery were validated by performing classification on original matrices, matrices with random missing values, and imputed matrices of the CLL and AML dataset. Classification can be either supervised or unsupervised depending on the availability of ground truth labels. In these dataset, ground truth labels were available. Hence, supervised classification was performed to distinguish between two classes, normal and cancer using two different classifiers: linear Support Vector Machine (SVM) and *k* nearest neighbor (KNN) method with *k* = 3. Both the dataset had large number of features, therefore, feature reduction was performed to extract important features from the data. Three different methods of feature reduction were used, Mutual Information criterion, Principal Component Analysis (PCA) and Chi-square method. Optimal number of features in each method were estimated by grid search. Further, five-fold cross validation was performed and average accuracy over 20 iterations was reported. Experiments were performed in Python 3 environment with Sklearn 0.20 library. Classification code was written in Python programming language. Scikit-learn is a Python module for machine learning and contains various algorithms related to regression, classification, and clustering. Examples of these algorithms are support vector machines (SVM), random forest (RF), *k*-means. Classification accuracy and *F*_1_ score were calculated at different sampling ratios from 10 to 90%. The accuracy and *F*_1_ score are defined as

(14) $$\text{Accuracy}=\frac{1}{M}\sum_{i=1}^{M}1(x_{i}={\tilde{x}}_{i}),$$

(15) $$\text{and }F_{1}=\frac{2\times \text{precision }\times \text{ recall}}{\text{precision }+\text{ recall}},$$

where *M* is the total number of samples in the dataset, *x*_*i*_ is the ground truth class label of the ith sample, and ${\tilde{x}}_{i}$ is the predicted class label. Weighted *F*_1_ score was used in order to account for label imbalance arising out due to unequal number of tumor and normal samples. CLL dataset had 188 tumor and 32 normal samples and AML dataset had 26 tumor samples and 38 normal. Tables 2, 3 clearly indicate that values of classification accuracy and *F*_1_ scores for incomplete matrices are low as compared to the values obtained on imputed matrices. Classification accuracy and *F*_1_ scores were also computed on imputed matrices obtained from the three existing methods on both the dataset and compared with the results of DSNN method as shown in Figures 5, 6. Classification was also performed on MM-Spanish dataset (Results are shown in Table T1). Classification could not be performed on MM-Indian data because it was a single class data, i.e., of tumor samples only.

**Table 2:** Classification accuracy and *F*_1_ score for CLL dataset at varying sampling ratios (FR, feature reduction; SR, sampling ratio; Obs., observed; Rec., recovered using DSNN method.

	**Classification accuracy**											
**FR**	**PCA**				**Chi-Square method**				**Mutual info method**			
	**KNN**		**Linear SVM**		**KNN**		**Linear SVM**		**KNN**		**Linear SVM**	
**SR**	**Obs**.	**Rec**.	**Obs**.	**Rec**.	**Obs**.	**Rec**.	**Obs**.	**Rec**.	**Obs**.	**Rec**.	**Obs**.	**Rec**.
10%	0.71	0.87	0.73	0.77	0.84	0.96	0.85	0.97	0.86	0.96	0.89	0.98
20%	0.71	0.87	0.75	0.78	0.80	0.97	0.84	0.98	0.86	0.98	0.87	0.99
30%	0.79	0.89	0.77	0.81	0.85	0.97	0.84	0.98	0.85	0.99	0.87	0.99
40%	0.79	0.89	0.81	0.91	0.85	0.98	0.85	0.98	0.86	0.99	0.88	0.99
50%	0.80	0.89	0.85	0.97	0.86	0.99	0.85	0.98	0.88	0.99	0.90	0.99
60%	0.78	0.92	0.87	0.97	0.85	0.99	0.85	0.98	0.90	0.99	0.92	0.99
70%	0.83	0.90	0.90	0.97	0.86	0.99	0.86	0.98	0.93	0.99	0.96	0.99
80%	0.83	0.91	0.96	0.98	0.86	0.99	0.87	0.98	0.98	0.99	0.99	0.99
90%	0.85	0.91	0.97	0.97	0.87	0.98	0.91	0.98	0.99	0.99	0.99	0.99
	**F**_**1**_ **score**											
**FR**	**PCA**				**Chi-Square method**				**Mutual info method**			
	**KNN**		**Linear SVM**		**KNN**		**Linear SVM**		**KNN**		**Linear SVM**	
**SR**	**Obs**.	**Rec**.	**Obs**.	**Rec**.	**Obs**.	**Rec**.	**Obs**.	**Rec**.	**Obs**.	**Rec**.	**Obs**.	**Rec**.
10%	0.72	0.86	0.72	0.72	0.78	0.96	0.79	0.96	0.79	0.95	0.85	0.98
20%	0.72	0.85	0.74	0.72	0.77	0.97	0.78	0.98	0.79	0.98	0.81	0.98
30%	0.78	0.88	0.77	0.77	0.79	0.97	0.78	0.97	0.79	0.99	0.82	0.99
40%	0.78	0.86	0.80	0.90	0.79	0.98	0.79	0.98	0.79	0.99	0.85	0.99
50%	0.80	0.88	0.84	0.96	0.80	0.99	0.79	0.98	0.84	0.99	0.87	0.99
60%	0.78	0.90	0.86	0.96	0.79	0.99	0.79	0.98	0.88	0.99	0.90	0.99
70%	0.82	0.89	0.90	0.97	0.80	0.99	0.80	0.98	0.92	0.99	0.96	0.99
80%	0.82	0.90	0.96	0.98	0.80	0.98	0.82	0.98	0.98	0.99	0.99	0.99
90%	0.84	0.91	0.97	0.97	0.82	0.98	0.89	0.98	0.98	0.99	0.99	0.99

**Table 3:** Classification Accuracy and *F*_1_ score for AML dataset at varying sampling ratios (FR, feature reduction; SR, sampling ratio; Obs., observed; Rec., recovered using DSNN method).

	**Classification accuracy**											
**FR**	**PCA**				**Chi-Square method**				**Mutual information method**			
	**KNN**		**Linear SVM**		**KNN**		**Linear SVM**		**KNN**		**Linear SVM**	
**SR**	**Obs**.	**Rec**.	**Obs**.	**Rec**.	**Obs**.	**Rec**.	**Obs**.	**Rec**.	**Obs**.	**Rec**.	**Obs**.	**Rec**.
10%	0.55	0.84	0.54	0.83	0.60	0.86	0.86	0.96	0.76	0.91	0.96	0.98
20%	0.50	0.98	0.50	0.98	0.97	0.97	0.98	0.98	0.73	0.99	0.91	0.99
30%	0.45	0.99	0.45	0.99	0.97	0.97	0.98	0.98	0.76	1.0	0.91	0.99
40%	0.53	0.99	0.59	0.99	0.95	0.99	0.99	1.0	0.71	1.0	0.86	1.0
50%	0.54	0.98	0.56	0.99	0.96	0.96	0.99	0.99	0.77	1.0	0.83	0.99
60%	0.63	0.98	0.70	0.99	0.98	1.0	0.99	1.0	0.75	1.0	0.93	1.0
70%	0.63	0.96	0.67	0.99	0.98	0.98	0.99	1.0	0.82	1.0	0.96	0.99
80%	0.75	0.96	0.77	0.99	0.99	0.99	0.96	1.0	0.87	0.98	0.96	1.0
90%	0.80	0.94	0.87	0.99	0.99	0.99	0.96	0.99	0.94	0.99	0.97	0.99
	**F**_**1**_ **score**											
**FR**	**PCA**				**Chi-Square method**				**Mutual information method**			
	**KNN**		**Linear SVM**		**KNN**		**Linear SVM**		**KNN**		**Linear SVM**	
**SR**	**Obs**.	**Rec**.	**Obs**.	**Rec**.	**Obs**.	**Rec**.	**Obs**.	**Rec**.	**Obs**.	**Rec**.	**Obs**.	**Rec**.
10%	0.53	0.83	0.54	0.83	0.48	0.85	0.86	0.95	0.76	0.91	0.96	0.98
20%	0.49	0.98	0.50	0.98	0.97	0.97	0.98	0.98	0.73	1.0	0.91	0.99
30%	0.45	1.0	0.46	0.99	0.97	0.97	0.98	0.99	0.76	1.0	0.91	0.99
40%	0.52	0.99	0.60	1.0	0.96	0.99	1.0	1.0	0.72	1.0	0.86	1.0
50%	0.53	0.98	0.57	0.99	0.96	0.96	0.99	0.99	0.78	1.0	0.82	0.99
60%	0.64	0.97	0.70	0.99	0.98	1.0	0.99	1.0	0.75	1.0	0.93	1.0
70%	0.64	0.97	0.68	1.0	0.98	0.98	0.99	1.0	0.82	1.0	0.96	0.99
80%	0.73	0.96	0.77	0.99	0.98	0.99	0.96	1.0	0.87	0.98	0.96	1.0
90%	0.77	0.93	0.87	0.98	0.99	0.99	0.96	0.99	0.94	0.99	0.97	0.99

#### 3.2.2. Experiment 2: Biological Validation

For biological validation of the results, SPARROW was applied on the original matrix, incomplete matrices, and imputed matrices to identify top candidate tumor driver genes. SPARROW (SPARse selected expRessiOn regulators identified With penalized regression) was proposed by Logsdon et al. (28) and aims to find out candidate tumor drivers from the “selected expression regulators” (SERs). It defines SERs as the genes that drive dysregulated transcription leading to carcinogenesis. In this method, variational Bayesian spike regression model has been used to fit the following model,

(16) $$y_{m,n}=\sum x_{m,k}\beta_{k,n}+e_{m,n},$$

where *y*_*m,k*_ is the value of expression of the *n*th gene for the *m*th subject, *e*_*m,n*_ is a normally distributed error, *x*_*m,k*_ is the value of expression of the *k*th SER for the *m*th subject and β_*k,n*_ is the additive effect of the expression of the *k*th SER on the expression of the *n*th gene. *m* ranges from 1….*M*, where *M* is the total number of subjects and *n* ranges from 1…….*N*, where *N* is the total number of genes. Total SERs used in the analysis were around 3,400 and they were downloaded from the link provided in the original paper. This algorithm provides a rank to each SER based on the gene expression values of the samples. The top-ranked genes from the list can be further studied by gene enrichment analysis.

For finding top 500 candidate driver genes, only the tumor samples from the data matrices were considered for SPARROW analysis. Algorithm was applied on original complete data matrices of all the dataset to identify the top-ranked candidate tumor drivers. This served as the ground truth for our analysis. Further, SPARROW was applied on incomplete and imputed data matrices of both the dataset at sampling ratios of 50 and 70%. Top-ranked candidate drivers from the incomplete and imputed data matrices were obtained. Gene enrichment analysis was performed on top 500 genes. KEGG and GO pathways were studied using web based application, Enrichr, developed, and maintained by Chen et al. (34) and Kuleshov et al. (35). KEGG pathways obtained from gene lists of original dataset were the ground truth. It was observed that when KEGG pathway analysis was done for incomplete matrices, these were not able to predict cancer pathways with a higher significance (low *p*-value) whereas for imputed matrices, cancer pathways were predicted with a higher significance due to decrease in *p*-value. Results from KEGG analysis on all dataset are presented in tabular form showing the *p*-values, combined score for original data and the incomplete and complete matrices in tabular form in Tables T2–T5. *p*-value was computed from the Fisher exact test. Fisher test was run on random gene sets and ranks were derived at each run. Mean rank was calculated from the different runs and standard deviation of the rank obtained from the expected rank was also calculated for each term in the gene-set library. Finally, a *z*-score was calculated to estimate the deviation from the expected rank. *z*-score and *p*-value were used to compute combined score which is obtained by multiplying z-score with the logarithm of *p*-value. A detailed analysis for CLL dataset consisting of *z*-score and combined score has also been shown in Tables T6, T7. Similarly, GO pathways were obtained from the Enrichr and bubble plots were constructed from GO tables as shown in Figures S5–S12.

## 4. Discussion

### 4.1. Importance of the Proposed DSNN Method

DSNN, a two stage method proposed for matrix recovery in the paper, was based on Compressive Sensing Framework. In Stage-1, it utilized column and row sparsity of the gene expression matrix in DCT domain for missing value imputation, while in Stage-2, it exploited low rank nature of the matrix for denoising. Expression values of any particular gene would vary slowly across subjects, thereby, exhibiting sparsity in columns in some transformed domain. Similarly, expression values of a subject for most of the genes will also be slowly varying, thereby, exhibiting sparsity in the rows. Since there is a high inter-dependence between the expression levels of the genes, one may consider gene expression matrix as a low rank matrix. Thus, as discussed earlier, both the assumptions used in Stage-1 (of sparsity in DCT domain) and Stage-2 (low rank of matrix) hold true for the given gene expression data. The concept of DCT-based sparsity was recently applied on biological data in methods, TV-DCT (26) and CT-NNBI (27), although only column sparsity in the DCT domain was used. On the other hand, this work utilizes double sparsity, i.e., sparsity on both the columns and the rows. Most of the imputation algorithms developed for missing value imputation such as KNN, LSimpute, LLSimpute, BPCA etc. work at high observability of data, while the proposed DSNN method worked well even when data had very high missing rates of 10–40%. The proposed DSNN method performed better than the other matrix completion methods at all sampling ratios. The state-of-the-art matrix imputation methods that have been used for performance comparison in this work required a lot of parameter tuning for optimal performance, while DSNN method did not require parameter tuning to such a great extent.

### 4.2. Improvement in Classification Accuracy

It was evident from the results shown in Tables 2, 3 that the classification accuracy and *F*_1_ scores reduced as the number of missing values increased. There were 220 samples in CLL dataset and 64 samples in AML dataset. For smaller dataset like AML, missing values affected the classification accuracy and *F*_1_ scores greatly. Thus, it is necessary to impute missing values in gene expression data to prevent incorrect downstream analysis of the data. When the classification was performed on the imputed data, there was considerable improvement in the classification accuracy, thereby, validating our hypothesis. Classification accuracy and *F*_1_ scores calculated on original complete data matrices (100% sampling ratio) were considered as ground truth values. For CLL dataset, ground truth values of classification accuracy and weighted *F*_1_ score were 0.99 and 0.99, respectively, as shown in Figure 5. For KNN classifier and Chi-square feature selection approach, classification accuracy and *F*_1_ score obtained for 50% observed data was 0.86 and 0.80, respectively as shown in Table 2. After imputation, values improved significantly to 0.99 and 0.99. For AML dataset, ground truth values of classification accuracy and *F*_1_ score were 1.0 and 1.0, respectively as shown in Figure 6. Similarly for Linear SVM classifier and PCA feature selection approach, classification accuracy and *F*_1_ score for 50% observed data was 0.56 and 0.57, respectively, as shown in Table 3. After matrix imputation, classification accuracy and *F*_1_ score improved considerably to 0.99 and 0.99, respectively. For every sampling ratio, consistent results were obtained that validates our method.

### 4.3. Improvement in Functional Enrichment Analysis for KEGG Pathways

**Figure 7:**
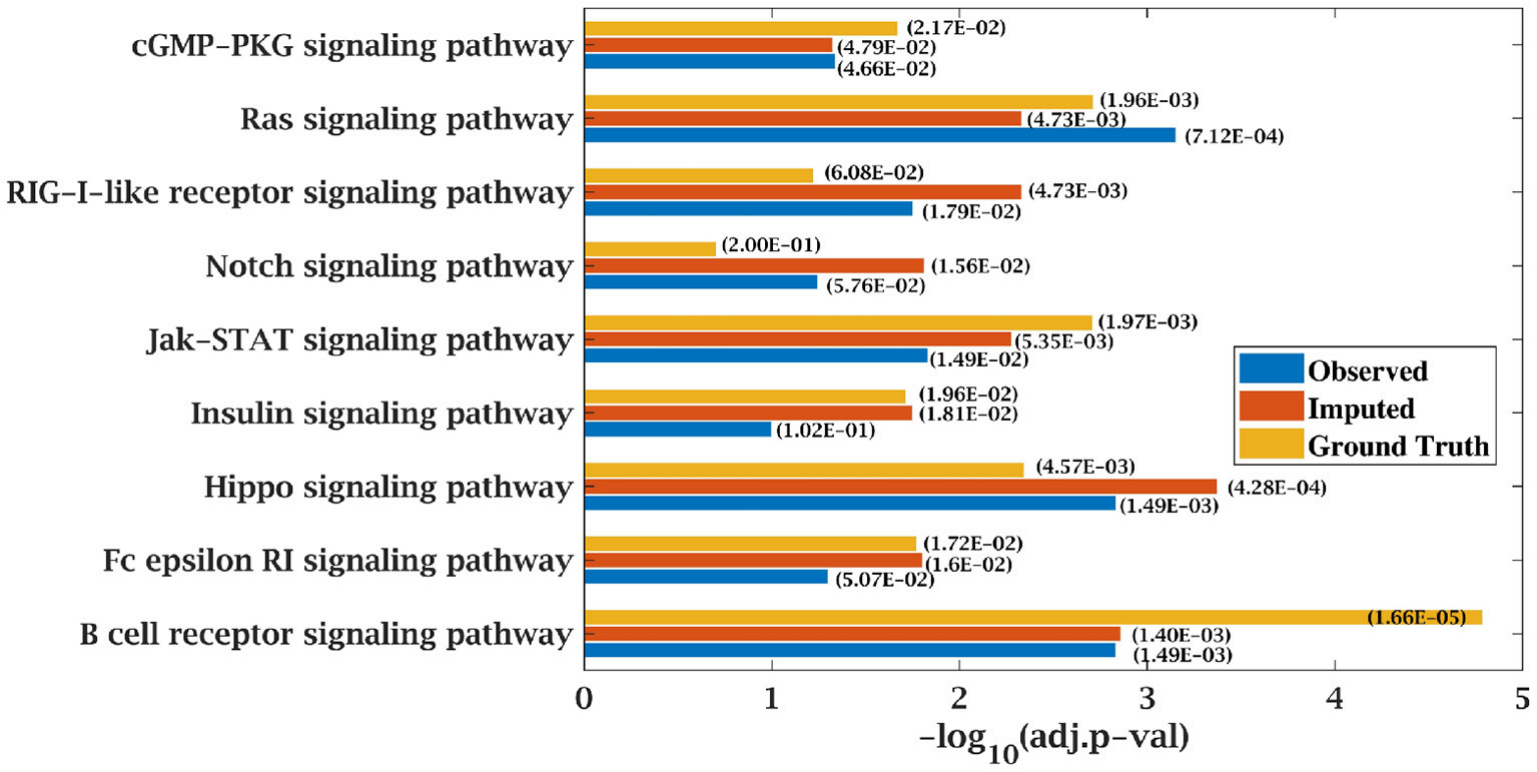
Few important KEGG pathways at 70% observed and imputed data for CLL data. Adjusted *p*-values are shown in brackets.

KEGG and GO enrichment analysis was performed on the top 500 ranked genes obtained from SPARROW algorithm to biologically validate our results. As mentioned earlier, KEGG pathways obtained by the top-ranked genes of original matrices were considered the ground truth values. Pathways with *p*-value < 0.05 were only considered. When KEGG analysis was done on top-ranked genes from incomplete matrices, there was significant decrease in the *p*-value of the most significant pathways. “Wnt signaling pathway” (41, 42) and “Notch signaling pathway” (43, 44) are important pathways in CLL cancer. An important observation was that *p*-value for “Notch signaling pathway” was 2.00E-01 at ground truth and it was 5.76E-02 at 70% observed data for CLL dataset. Values were insignificant in both the cases. However, after imputation, *p*-value became significant with value 1.56E-02 which was <0.05 as shown in Figure 7.

**Figure 8:**
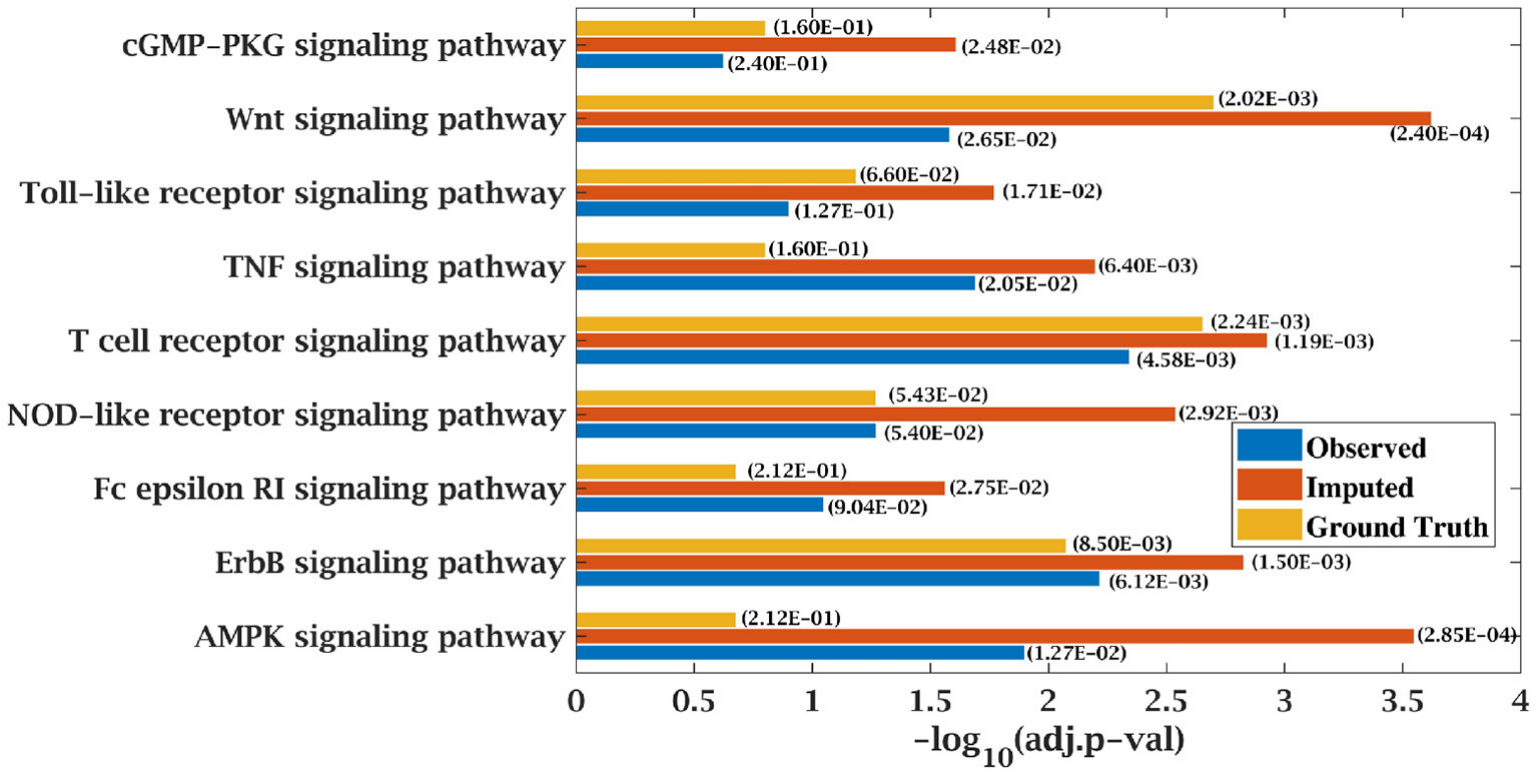
Few important KEGG pathways at 70% observed and imputed data for AML data. Adjusted *p*-values are shown in brackets.

**Figure 9:**
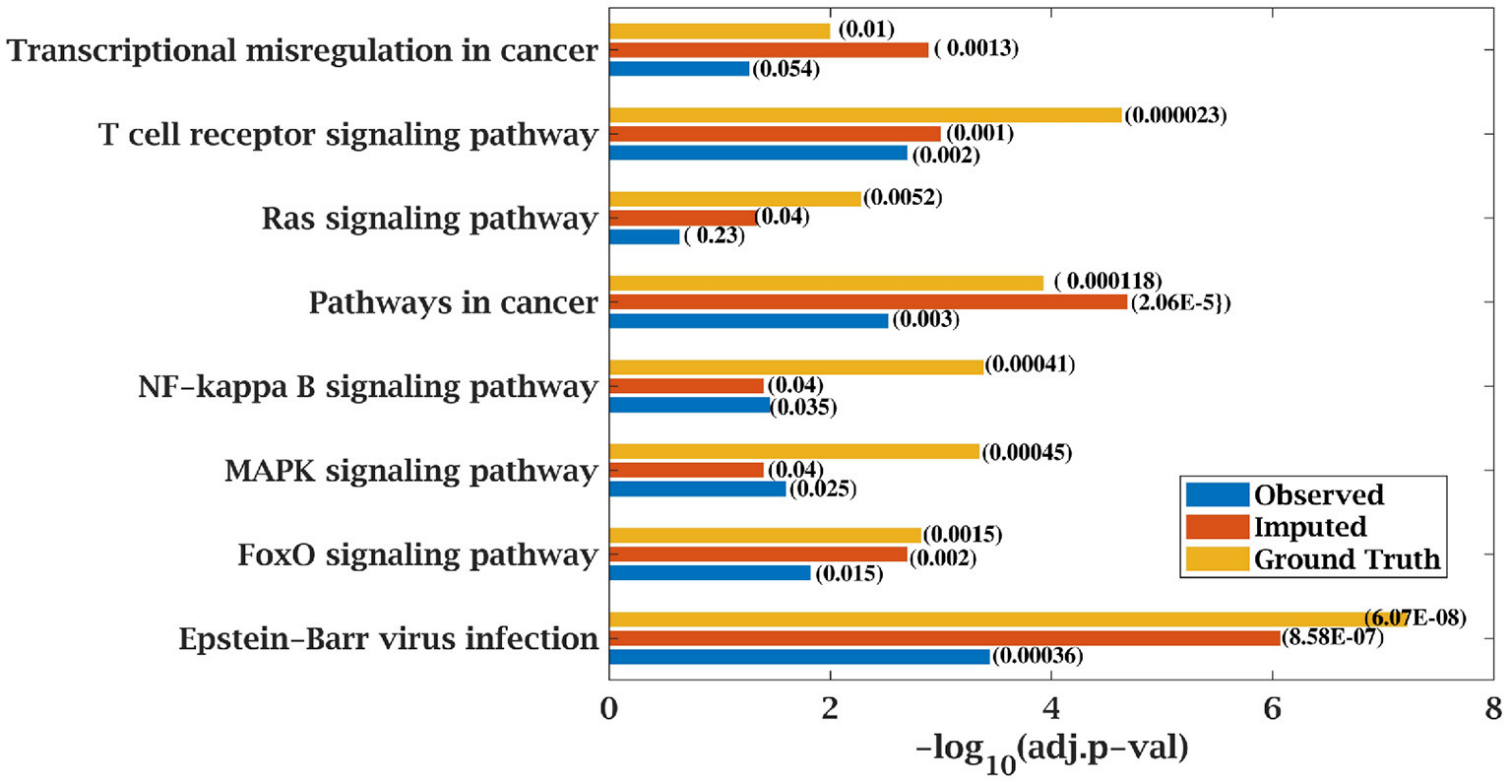
Few important KEGG pathways at 70% observed and imputed data for MM-Spanish data. Adjusted *p*-values are shown in brackets.

**Figure 10:**
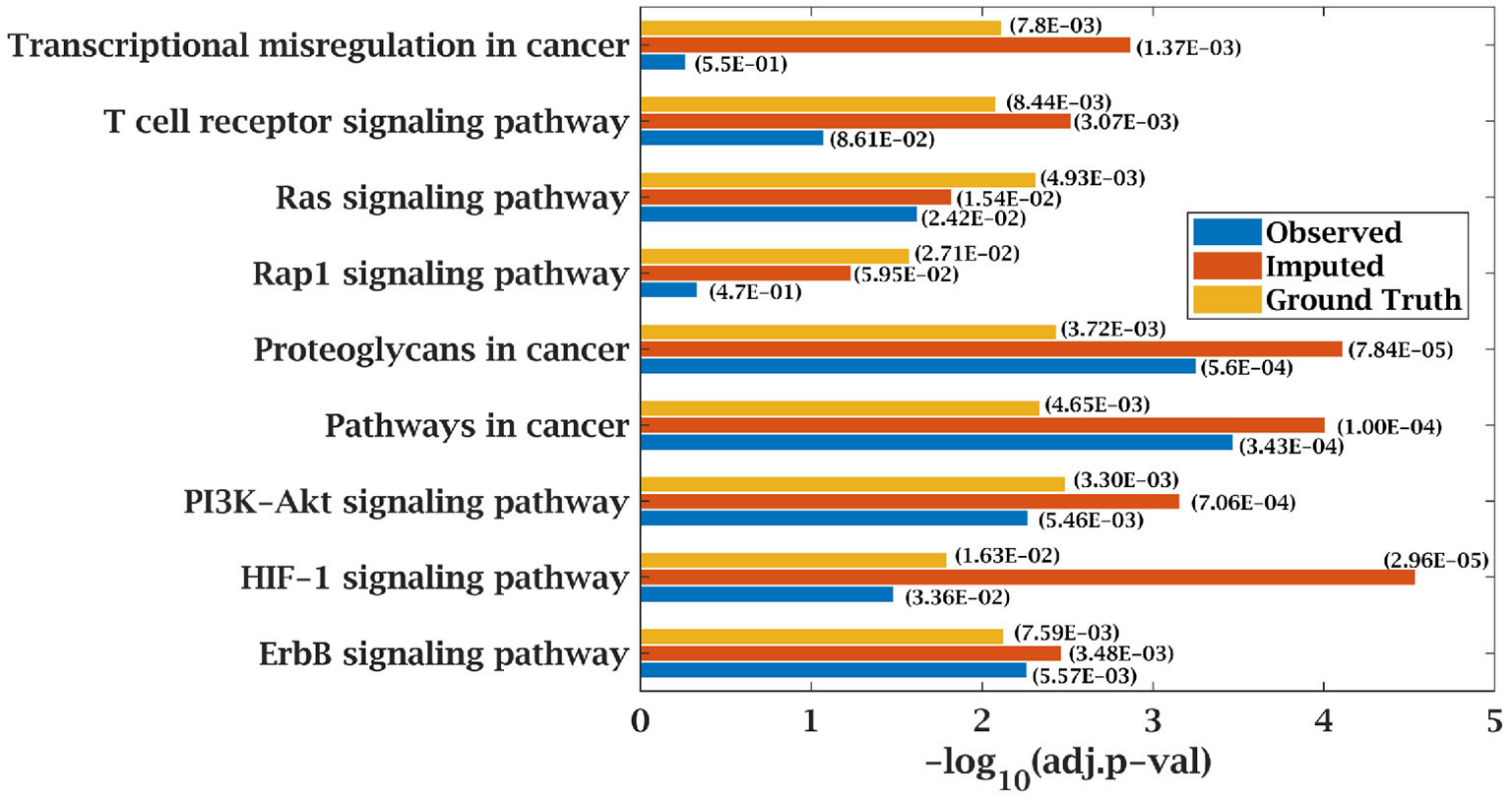
Few important KEGG pathways at 70% observed and imputed data for MM-Indian data. Adjusted *p*-values are shown in brackets.

Similarly, *p*-value for “Wnt signaling pathway” was 8.33E-05 on original dataset, as shown in Figure S1. At 50% observed data p-value for “Wnt signaling pathway” was 3.10E-02 which was less significant than the ground truth value at 50% observed data. After matrix imputation, *p*-value became significant with value 2.13E-03. Similarly, *p*-value became 1.90E-05 after matrix imputation on 70% observed data which was more significant than the *p*-value 6.66E-5, observed at 70% data. “Fc epsilon RI signaling pathway” is an important pathway in AML cancer (45). This pathway was insignificant for original data with *p*-value-2.12E-01. At 70% observed data, *p*-value was 9.40E-02 which was again greater than 0.05. After matrix imputation, the value became significant at 2.75E-02, which was less than 0.05 as shown in Figure 8. Similarly, ‘Ras signaling pathway” is activated in Multiple Myeloma cancer (46). For MM-Spanish data, “Ras signaling pathway” was significant with *p*-value-0.0052 for original data but became insignificant with *p*-value-0.23 when 70% data was observed as shown in Figure 9. After matrix imputation, significance of the pathway was restored with *p*-value 0.04. For MM-Indian dataset,“Transcriptional misregulation in cancer” was found to be insignificant with *p*-value 0.47 as shown in Figure 10. After imputation, *p*-value decreased to 1.37E-03 and became more significant than ground truth *p*-value, 7.8E-03. Additional KEGG analysis results on the dataset CLL, AML and MM Spanish data are provided in the Figures S2–S4.

Thus, DSNN method not only imputed missing entries but also performed some denoising to improve the results. It is quite evident from the analysis that gene enrichment analysis results were partially inaccurate due to incomplete matrices. This was because the genes identified as top-ranked genes by performing SPARROW analysis on complete data matrix were not identified in the top-ranked list obtained from incomplete data matrix. However, when the incomplete matrix was imputed using the proposed DSNN method, top-ranked list of genes obtained from SPARROW analysis was quite similar to the ground truth. Our observations demonstrate the importance of imputing missing values in gene expression data.

## 5. Conclusion

Microarray data generally has a lot of missing values that can adversely influence the downstream analysis. In this paper, a new method, namely DSNN, is proposed that imputes missing values in the gene expression data using discrete cosine transform based double sparsity and nuclear norm minimization. Method was also validated quantitatively based on the application of classification approach as well as biologically by performing pathway enrichment analysis and showed consistent findings.

## Data Availability Statement

Data was downloaded from GEO database with accession numbers, GSE9476, GSE50006, and GSE47552 and GSE125361. Code of the DSNN method proposed in this manuscript is available publicly at github:https://github.com/AkankshaFarswan/DSNN_MatrixImputation.

## Author Contributions

AF and AG conceptualized the method of data imputation. RG and GK contributed to the ideas on method application and validation in bioinformatics. AF prepared all codes, generated results, and prepared the first draft of the paper. All authors edited the manuscript for final submission.

### Conflict of Interest

The authors declare that the research was conducted in the absence of any commercial or financial relationships that could be construed as a potential conflict of interest.
