# Supplementary material for: Imputation of Gene Expression Data in Blood Cancer and Its Significance in Inferring Biological Pathways
Source: Front Oncol. 2020 Jan 8;9:1442. doi: 10.3389/fonc.2019.01442 (PMC6960109; doi:10.3389/fonc.2019.01442)
Supplement: Supplementary file 1 [file Data_Sheet_1.pdf]

Supplementary Material to "Imputation of gene expression data in blood cancer and its significance in inferring biological pathways"

1 SUPPLEMENTARY TABLES AND FIGURES

This Section contains additional results in the form of tables and bubble plots on the four dataset- CLL, AML, MM-Spanish and MM-India. It contains results obtained from KEGG pathway analysis and GO analysis. These tables and figures are appropriately referred in the main manuscript.

Table-T1: Classification accuracy and F_1 scores on different sampling percentage of incomplete matrix and the recovered/imputed matrix on MM-Spanish data.
SR stands for Sampling ratio of observed data to the total data (in percentage).

	**Classification Accuracy**				**F_1 score**			
	**KNN**		**Linear SVM classifier**		**KNN**		**Linear SVM classifier**	
SR	Observed	Imputed	Observed	Imputed	Observed	Imputed	Observed	Imputed
10	0.22	**0.60**	0.77	**0.80**	0.21	**0.59**	0.75	**0.80**
20	0.35	**0.82**	0.70	**0.88**	0.37	**0.82**	0.68	**0.88**
30	0.60	**0.88**	0.72	**0.90**	0.60	**0.88**	0.71	**0.90**
40	0.56	**0.86**	0.65	**0.92**	0.54	**0.84**	0.64	**0.92**
50	0.54	**0.84**	0.72	**0.94**	0.65	**0.84**	0.70	**0.94**
60	0.66	**0.83**	0.76	**0.93**	0.63	**0.84**	0.74	**0.93**
70	0.63	**0.89**	0.75	**0.92**	0.62	**0.89**	0.74	**0.92**
80	0.67	**0.86**	0.83	**0.92**	0.86	**0.86**	0.81	**0.92**
90	0.67	**0.87**	0.86	**0.94**	0.88	**0.87**	0.85	**0.94**

Table-T2: Adjusted p-values for KEGG pathways at ground truth, 50% observed and imputed data, 70% observed and imputed data for CLL dataset.

		50%		70%	
**Term**	**Ground Truth**	**Observed**	**Recovered**	**Observed**	**Recovered**
Pathways in cancer	2.75E-13	6.17E-07	1.08E-08	1.21E-11	5.59E-13
Transcriptional misregulation in cancer	3.30E-15	3.25E-15	1.82E-12	3.26E-15	6.98E-12
MAPK signaling pathway	3.13E-08	5.71E-06	1.50E-05	5.13E-12	5.25E-10
Acute myeloid leukemia	3.04E-08	3.31E-03	1.57E-04	3.01E-08	2.79E-07
T cell receptor signaling pathway	2.55E-08	2.28E-01	2.25E-01	2.40E-04	1.48E-05
Chronic myeloid leukemia	5.54E-08	9.35E-03	3.88E-05	3.44E-07	7.84E-09
HTLV-I infection	1.42E-07	5.35E-05	5.22E-07	5.13E-12	6.34E-07
Proteoglycans in cancer	5.69E-07	1.51E-03	1.85E-03	2.21E-05	8.40E-06
ErbB signaling pathway	2.56E-06	1.51E-01	2.73E-03	5.51E-05	5.09E-05
B cell receptor signaling pathway	1.66E-05	2.14E-01	2.11E-01	1.49E-03	1.40E-03
AGE-RAGE signaling pathway	5.93E-05	4.02E-02	1.70E-02	1.91E-04	4.35E-05
Neurotrophin signaling pathway	6.74E-05	4.82E-03	3.88E-05	1.27E-07	3.38E-06
Wnt signaling pathway	8.33E-05	3.10E-02	2.13E-03	6.66E-05	1.90E-05
Signaling pathways regulating pluripotency of stem cells	8.33E-05	6.01E-04	3.74E-06	5.33E-06	6.18E-05
Chemokine signaling pathway	1.13E-04	2.02E-01	2.47E-02	3.82E-02	8.19E-02
TNF signaling pathway	1.13E-04	2.81E-04	2.25E-02	1.94E-07	1.27E-06
TGF-beta signaling pathway	2.37E-04	1.27E-04	4.75E-04	4.27E-05	1.81E-04
Toll-like receptor signaling pathway	3.17E-04	6.52E-03	1.14E-01	1.57E-05	3.99E-06
PI3K-Akt signaling pathway	1.43E-03	2.71E-03	2.14E-02	3.31E-05	1.99E-04
p53 signaling pathway	2.67E-04	2.23E-03	2.47E-02	2.22E-04	9.81E-04
HIF-1 signaling pathway	1.02E-03	6.00E-03	1.82E-02	1.24E-05	1.99E-04
NF-kappa B signaling pathway	4.65E-04	1.48E-05	3.53E-03	9.93E-05	2.19E-05
Ras signaling pathway	1.96E-03	3.82E-02	3.99E-03	7.12E-04	4.73E-03
Jak-STAT signaling pathway	1.97E-03	2.03E-01	2.02E-01	1.49E-02	5.35E-03
Hippo signaling pathway	4.57E-03	1.10E-03	3.99E-04	1.49E-03	4.28E-04
FoxO signaling pathway	5.12E-03	5.27E-02	2.25E-02	1.56E-03	3.59E-05
Phospholipase D signaling pathway	8.76E-03	6.70E-01	1.50E-01	1.16E-01	5.33E-02
RNA degradation	2.04E-03	3.99E-03	1.45E-02	1.96E-03	8.58E-07
cGMP-PKG signaling pathway	2.17E-02	7.95E-01	7.87E-01	4.66E-02	4.79E-02
Insulin signaling pathway	1.96E-02	6.49E-01	6.39E-02	1.02E-01	1.81E-02
cAMP signaling pathway	5.82E-02	5.60E-01	2.36E-01	1.05E-02	5.33E-02
NOD-like receptor signaling pathway	1.74E-03	3.31E-03	1.21E-01	1.56E-03	1.18E-05
Fc epsilon RI signaling pathway	1.72E-02	3.98E-01	1.83E-01	5.07E-02	1.60E-02
RIG-I-like receptor signaling pathway	6.08E-02	2.02E-01	7.16E-01	1.79E-02	4.73E-03
Notch signaling pathway	2.00E-01	2.95E-04	6.94E-03	5.76E-02	1.56E-02

Table-T3: Adjusted p-values for KEGG pathways at ground truth, 50% observed and imputed data, 70% observed and imputed data for AML dataset.

		50%		70%	
**Term**	**Ground Truth**	**Observed**	**Recovered**	**Observed**	**Recovered**
Signaling pathways regulating pluripotency of stem cells	2.76E-12	2.06E-19	1.63E-17	1.57E-15	1.02E-12
Pathways in cancer	2.71E-09	6.56E-10	8.63E-06	2.81E-11	2.81E-11
HTLV-I infection	1.58E-05	5.59E-07	2.86E-04	2.34E-06	2.34E-06
Hippo signaling pathway	9.62E-06	2.21E-06	3.11E-05	2.99E-07	7.10E-06
Transcriptional misregulation in cancer	1.58E-05	5.42E-04	1.37E-07	2.71E-08	2.71E-08
TGF-beta signaling pathway	2.47E-05	9.89E-07	1.15E-06	2.30E-09	1.15E-06
FoxO signaling pathway	1.21E-04	6.34E-06	4.98E-02	6.27E-06	1.23E-06
Proteoglycans in cancer	6.57E-04	7.41E-05	1.89E-04	2.00E-07	2.00E-07
Hedgehog signaling pathway	4.57E-04	7.41E-05	1.48E-03	2.31E-04	8.04E-03
Cytokine-cytokine receptor interaction	1.33E-03	1.27E-02	7.11E-03	1.09E-03	3.57E-06
p53 signaling pathway	6.57E-04	5.56E-04	1.66E-03	1.66E-03	5.31E-05
PI3K-Akt signaling pathway	2.97E-03	2.53E-04	1.02E-03	1.02E-03	5.26E-07
Wnt signaling pathway	2.02E-03	5.88E-04	8.98E-04	2.65E-02	2.40E-04
T cell receptor signaling pathway	2.24E-03	2.36E-03	4.64E-02	4.58E-03	1.19E-03
Jak-STAT signaling pathway	4.38E-03	1.61E-04	6.49E-03	6.36E-04	6.36E-04
HIF-1 signaling pathway	6.72E-03	1.61E-04	5.36E-05	4.32E-03	1.02E-05
ErbB signaling pathway	8.50E-03	2.30E-02	6.12E-03	6.12E-03	1.50E-03
Neurotrophin signaling pathway	1.67E-02	8.99E-02	8.08E-02	1.06E-02	1.06E-02
MAPK signaling pathway	2.47E-02	3.26E-04	1.23E-02	2.49E-04	8.05E-05
Ras signaling pathway	4.98E-02	1.89E-02	2.09E-01	6.22E-04	4.70E-06
Toll-like receptor signaling pathway	6.60E-02	1.33E-01	2.74E-01	1.27E-01	1.71E-02
AGE-RAGE signaling pathway	1.20E-01	5.35E-03	7.22E-01	3.83E-03	4.47E-05
cGMP-PKG signaling pathway	1.60E-01	4.41E-01	7.91E-01	2.40E-01	2.48E-02
TNF signaling pathway	1.60E-01	1.48E-01	7.65E-01	2.05E-02	6.40E-03
Rap1 signaling pathway	2.00E-01	1.99E-01	4.33E-01	7.02E-03	2.42E-05
NOD-like receptor signaling pathway	5.43E-02	1.40E-01	4.19E-01	5.40E-02	2.92E-03
AMPK signaling pathway	2.12E-01	5.91E-03	3.98E-03	1.27E-02	2.85E-04
Fc epsilon RI signaling pathway	2.12E-01	2.54E-02	5.10E-01	9.04E-02	2.75E-02

Table-T4: Adjusted p-values for KEGG pathways at ground truth, 50% observed and imputed data, 70% observed and imputed data for MM-Spanish dataset.

		50%		70%	
**Term**	**Ground Truth**	**Observed**	**Recovered**	**Observed**	**Recovered**
Epstein-Barr virus infection	6.07E-08	8.04E-03	2.16E-03	3.61E-04	8.59E-07
T cell receptor signaling pathway	2.31E-05	6.10E-02	2.76E-02	2.34E-03	1.41E-03
Pathways in cancer	1.18E-04	1.92E-04	5.94E-09	3.37E-03	2.07E-05
RNA polymerase	4.92E-05	3.32E-01	3.43E-01	3.47E-01	3.84E-02
RNA degradation	1.18E-04	5.45E-02	4.26E-04	4.60E-02	7.65E-03
MAPK signaling pathway	4.50E-04	1.40E-01	2.81E-02	2.58E-02	4.67E-02
NF-kappa B signaling pathway	4.10E-04	5.83E-01	9.61E-02	3.57E-02	4.04E-02
FoxO signaling pathway	1.59E-03	6.95E-02	1.82E-03	1.54E-03	2.56E-03
Apoptosis	2.22E-03	7.94E-01	3.99E-02	7.59E-02	1.67E-02
Neurotrophin signaling pathway	2.22E-03	9.39E-02	2.25E-03	4.08E-02	4.51E-02
HTLV-I infection	2.62E-03	9.13E-03	2.36E-01	8.16E-03	1.18E-03
Ras signaling pathway	5.26E-03	1.46E-01	2.81E-02	2.37E-01	4.67E-02
Rap1 signaling pathway	6.37E-03	3.65E-02	3.36E-02	1.83E-01	5.83E-02
Thyroid hormone signaling pathway	6.22E-03	2.80E-03	2.16E-03	5.64E-04	1.25E-03
PI3K-Akt signaling pathway	6.62E-03	1.91E-02	4.02E-03	3.37E-03	1.58E-02
AGE-RAGE signaling pathway in diabetic complications	6.59E-03	2.65E-02	2.38E-02	1.07E-01	9.55E-03
VEGF signaling pathway	6.25E-03	1.58E-01	6.69E-02	1.55E-01	1.53E-01
RNA transport	7.73E-03	2.74E-01	1.66E-01	8.34E-02	1.07E-02
HIF-1 signaling pathway	6.89E-03	1.20E-01	9.45E-03	2.50E-02	2.31E-01
Transcriptional misregulation in cancer	1.03E-02	8.04E-03	2.91E-04	5.41E-02	1.33E-03
Fc epsilon RI signaling pathway	8.37E-03	6.67E-01	9.37E-02	1.94E-01	1.94E-01
Signaling pathways regulating pluripotency of stem cells	1.43E-02	5.00E-03	8.24E-03	3.57E-03	9.28E-03
Prolactin signaling pathway	1.09E-02	4.33E-01	4.07E-02	3.68E-02	4.16E-02
ErbB signaling pathway	2.54E-02	1.58E-01	3.08E-02	3.96E-03	1.12E-02
cAMP signaling pathway	4.16E-02	5.00E-03	3.50E-05	2.50E-02	4.67E-02
B cell receptor signaling pathway	3.66E-02	2.26E-01	4.23E-02	9.69E-02	1.04E-01

Table-T5: Adjusted p-values for KEGG pathways at ground truth and 70% observed and imputed data for MM-Indian dataset.

		70%	
**Term**	**Adjusted P-value**	**Observed**	**Recovered**
Signaling pathways regulating pluripotency of stem cells	3.23E-03	3.31E-04	2.26E-05
Pathways in cancer	4.65E-03	3.43E-04	1.00E-04
PI3K-Akt signaling pathway	3.30E-03	5.46E-03	7.06E-04
Proteoglycans in cancer	3.72E-03	5.68E-04	7.84E-05
Ras signaling pathway	4.93E-03	2.42E-02	1.54E-02
Breast cancer	4.33E-03	4.45E-04	1.53E-03
Gastric cancer	4.26E-03	6.27E-05	8.14E-05
T cell receptor signaling pathway	8.44E-03	8.61E-02	3.07E-03
Non-small cell lung cancer	7.95E-03	5.50E-03	1.12E-02
Transcriptional misregulation in cancer	7.82E-03	5.55E-01	1.37E-03
ErbB signaling pathway	7.59E-03	5.57E-03	3.48E-03
Regulation of actin cytoskeleton	8.59E-03	1.05E-01	1.36E-01
Spliceosome	1.31E-02	3.52E-04	1.51E-02
Pancreatic cancer	1.23E-02	2.89E-03	1.76E-02
Herpes simplex virus 1 infection	1.41E-02	5.43E-03	5.48E-04
Human papillomavirus infection	1.33E-02	1.02E-02	1.53E-03
HIF-1 signaling pathway	1.63E-02	3.36E-02	2.96E-05
Hepatocellular carcinoma	1.86E-02	3.17E-04	5.62E-04
Acute myeloid leukemia	2.07E-02	1.64E-02	7.61E-04
Colorectal cancer	2.14E-02	2.17E-03	3.30E-04
Renal cell carcinoma	2.43E-02	1.96E-02	3.48E-04
TGF-beta signaling pathway	2.49E-02	5.52E-02	9.17E-02
Rap1 signaling pathway	2.71E-02	4.73E-01	5.95E-02
Melanoma	2.62E-02	3.84E-01	1.52E-02
Primary immunodeficiency	2.55E-02	6.56E-01	5.53E-02
Cellular senescence	2.67E-02	3.84E-01	7.30E-03
Thyroid hormone signaling pathway	2.80E-02	1.36E-01	4.13E-02
Chronic myeloid leukemia	3.07E-02	9.44E-03	1.82E-02
Prostate cancer	3.06E-02	4.02E-03	7.19E-03
Sphingolipid signaling pathway	3.01E-02	6.91E-02	3.43E-04
Cell cycle	3.83E-02	1.67E-01	1.21E-01

	100%	50%		70%	
	Ground Truth	Observed	Imputed	Observed	Imputed
**1**	**Transcriptional misregulation in cancer_Homo sapiens_hsa05202**				
**Overlap**	31/180	31/180	28/180	31/180	27/180
**Adj. p-value**	3.30E-15	3.25E-15	1.82E-12	3.26E-15	6.98E-12
**Comb. score**	67.49	67.49	56.44	67.49	52.19
**2**	**Pathways in cancer_Homo sapiens_hsa05200**				
**Overlap**	42/397	32/397	35/397	39/397	42/397
**Adj. p-value**	2.75E-13	6.17E-07	1.08E-08	1.21E-11	5.59E-13
**Comb. score**	69.69	39.19	47.65	57.57	70.58
**3**	**Hepatitis B_Homo sapiens_hsa05161**				
**Overlap**	24/146	14/146	15/146	24/146	24/146
**Adj. p-value**	1.45E-11	2.80E-04	6.18E-05	1.08E-11	1.47E-11
**Comb. score**	54.83	18.06	20.59	54.09	54.83
**4**	**Measles_Homo sapiens_hsa05162**				
**Overlap**	23/136	10/136	8/136	15/136	16/136
**Adj. p-value**	1.86E-11	9.35E-03	5.86E-02	1.20E-05	3.38E-06
**Comb. score**	50.89	4.87	0.75	16.69	22.20
**5**	**Herpes simplex infection_Homo sapiens_hsa05168**				
**Overlap**	23/185	14/185	13/185	21/185	23/185
**Adj. p-value**	9.76E-09	1.83E-03	4.88E-03	1.27E-07	7.84E-09
**Comb. score**	37.11	10.24	7.75	28.19	36.58
**6**	**T cell receptor signaling pathway_Homo sapiens_hsa04660**				
**Overlap**	17/104	5/104	5/104	11/104	13/104
**Adj. p-value**	2.55E-08	2.28E-01	2.25E-01	2.40E-04	1.48E-05
**Comb. score**	36.46	-1.59	-1.76	6.47	15.99
**7**	**Acute myeloid leukemia_Homo sapiens_hsa05221**				
**Overlap**	13/57	7/57	9/57	13/57	12/57
**Adj. p-value**	3.04E-08	3.31E-03	1.57E-04	3.01E-08	2.79E-07
**Comb. score**	36.94	7.01	17.66	36.94	31.04
**8**	**MAPK signaling pathway_Homo sapiens_hsa04010**				
**Overlap**	26/255	23/255	22/255	32/255	29/255
**Adj. p-value**	3.13E-08	5.71E-06	1.50E-05	5.13E-12	5.25E-10
**Comb. score**	37.06	30.54	27.12	60.04	48.47
**9**	**Epstein-Barr virus infection_Homo sapiens_hsa05169**				
**Overlap**	23/202	18/202	18/202	21/202	25/202
**Adj. p-value**	3.13E-08	7.06E-05	5.87E-05	4.25E-07	1.81E-09
**Comb. score**	35.55	22.01	21.38	26.48	43.94
**10**	**Chronic myeloid leukemia_Homo sapiens_hsa05220**				
**Overlap**	14/73	7/73	11/73	13/73	15/73
**Adj. p-value**	5.54E-08	9.35E-03	3.88E-05	3.44E-07	7.84E-09
**Comb. score**	32.25	3.22	22.10	25.72	38.24
**11**	**HTLV-I infection_Homo sapiens_hsa05166**				
**Overlap**	25/258	21/258	25/258	32/258	24/258
**Adj. p-value**	1.42E-07	5.35E-05	5.22E-07	5.13E-12	6.34E-07
**Comb. score**	31.48	22.93	35.20	57.00	28.98
**12**	**Viral carcinogenesis_Homo sapiens_hsa05203**				
**Overlap**	22/205	14/205	11/205	24/205	18/205
**Adj. p-value**	1.61E-07	3.99E-03	4.05E-02	1.15E-08	2.61E-05
**Comb. score**	31.30	8.90	1.39	40.27	15.07
**13**	**Thyroid cancer_Homo sapiens_hsa05216**				
**Overlap**	9/29	6/29	9/29	6/29	9/29
**Adj. p-value**	3.26E-07	6.64E-04	1.06E-06	2.59E-04	4.31E-07
**Comb. score**	19.55	7.12	29.27	-10.53	22.79
**14**	**Proteoglycans in cancer_Homo sapiens_hsa05205**				
**Overlap**	21/203	15/203	15/203	18/203	19/203
**Adj. p-value**	5.69E-07	1.51E-03	1.85E-03	2.21E-05	8.40E-06
**Comb. score**	28.45	12.38	13.27	15.46	21.20
**15**	**Inflammatory bowel disease (IBD)_Homo sapiens_hsa05321**				
**Overlap**	12/65	11/65	11/65	13/65	14/65
**Adj. p-value**	7.84E-07	1.48E-05	1.50E-05	1.27E-07	1.39E-08
**Comb. score**	23.71	25.06	25.06	32.13	36.19
**16**	**Pancreatic cancer_Homo sapiens_hsa05212**				
**Overlap**	12/66	7/66	9/66	13/66	12/66
**Adj. p-value**	8.78E-07	6.48E-03	4.31E-04	1.27E-07	1.10E-06
**Comb. score**	23.89	5.03	14.86	31.33	25.70
**17**	**ErbB signaling pathway_Homo sapiens_hsa04012**				
**Overlap**	13/87	5/87	9/87	11/87	11/87
**Adj. p-value**	2.56E-06	1.51E-01	2.73E-03	5.51E-05	5.09E-05
**Comb. score**	21.71	-1.29	10.26	9.84	8.48
**18**	**Spliceosome_Homo sapiens_hsa03040**				
**Overlap**	16/134	16/134	13/134	15/134	13/134
**Adj. p-value**	2.56E-06	9.06E-06	3.99E-04	1.08E-05	1.35E-04
**Comb. score**	20.95	25.83	15.02	16.95	7.37
**19**	**Osteoclast differentiation_Homo sapiens_hsa04380**				
**Overlap**	15/132	14/132	11/132	17/132	13/132
**Adj. p-value**	1.09E-05	1.05E-04	3.38E-03	3.88E-07	1.18E-04
**Comb. score**	19.17	19.68	9.28	25.39	7.89
**20**	**Hepatitis C_Homo sapiens_hsa05160**				
**Overlap**	15/133	8/133	9/133	15/133	14/133
**Adj. p-value**	1.14E-05	5.27E-02	2.25E-02	1.06E-05	3.59E-05
**Comb. score**	18.38	0.78	2.68	18.03	12.18
**21**	**B cell receptor signaling pathway_Homo sapiens_hsa04662**				
**Overlap**	11/73	4/73	4/73	8/73	8/73
**Adj. p-value**	1.66E-05	2.14E-01	2.11E-01	1.49E-03	1.40E-03
**Comb. score**	17.46	-2.50	-2.73	1.12	-0.69
**22**	**Influenza A_Homo sapiens_hsa05164**				
**Overlap**	17/175	10/175	9/175	16/175	16/175
**Adj. p-value**	1.69E-05	3.91E-02	7.88E-02	4.70E-05	4.44E-05
**Comb. score**	18.71	1.93	0.10	12.99	12.09
**23**	**Colorectal cancer_Homo sapiens_hsa05210**				
**Overlap**	10/62	5/62	6/62	9/62	10/62
**Adj. p-value**	2.36E-05	5.35E-02	1.84E-02	1.05E-04	1.90E-05
**Comb. score**	14.51	-2.20	1.47	5.44	11.49
**24**	**Renal cell carcinoma_Homo sapiens_hsa05211**				
**Overlap**	10/66	7/66	8/66	12/66	9/66
**Adj. p-value**	4.06E-05	6.48E-03	2.13E-03	7.72E-07	1.52E-04
**Comb. score**	13.32	4.44	10.37	21.49	3.14
**25**	**AGE-RAGE signaling pathway in diabetic complications_Homo sapiens_hsa04933**				
**Overlap**	12/101	7/101	8/101	11/101	12/101
**Adj. p-value**	5.93E-05	4.02E-02	1.70E-02	1.91E-04	4.35E-05
**Comb. score**	15.76	1.49	4.95	8.62	12.41
**26**	**Legionellosis_Homo sapiens_hsa05134**				
**Overlap**	9/55	11/55	7/55	10/55	9/55
**Adj. p-value**	5.93E-05	5.71E-06	3.26E-03	8.55E-06	4.35E-05
**Comb. score**	10.66	29.69	7.40	15.56	5.72
**27**	**Neurotrophin signaling pathway_Homo sapiens_hsa04722**				
**Overlap**	13/120	10/120	14/120	17/120	15/120
**Adj. p-value**	6.74E-05	4.82E-03	3.88E-05	1.27E-07	3.38E-06
**Comb. score**	13.78	7.30	22.43	30.22	21.43
**28**	**Wnt signaling pathway_Homo sapiens_hsa04310**				
**Overlap**	14/142	9/142	12/142	14/142	15/142
**Adj. p-value**	8.33E-05	3.10E-02	2.13E-03	6.66E-05	1.90E-05
**Comb. score**	13.11	2.25	11.43	10.83	15.26
**29**	**Signaling pathways regulating pluripotency of stem cells_hsa04550**				
**Overlap**	14/142	13/142	17/142	16/142	14/142
**Adj. p-value**	8.33E-05	6.01E-04	3.74E-06	5.33E-06	6.18E-05
**Comb. score**	12.96	13.68	27.86	19.89	9.32
**30**	**Prostate cancer_Homo sapiens_hsa05215**				
**Overlap**	11/89	5/89	7/89	12/89	13/89
**Adj. p-value**	8.33E-05	1.59E-01	2.37E-02	1.47E-05	3.38E-06
**Comb. score**	12.36	-1.36	1.84	15.57	22.43
**31**	**Insulin resistance_Homo sapiens_hsa04931**				
**Overlap**	12/109	9/109	9/109	13/109	12/109
**Adj. p-value**	1.09E-04	7.23E-03	8.58E-03	2.06E-05	7.99E-05
**Comb. score**	11.64	4.96	5.98	13.78	8.17
**32**	**Chemokine signaling pathway_Homo sapiens_hsa04062**				
**Overlap**	16/187	8/187	11/187	10/187	9/187
**Adj. p-value**	1.13E-04	2.02E-01	2.47E-02	3.82E-02	8.19E-02
**Comb. score**	11.88	-0.82	2.32	-1.91	-2.45

Table-T6: KEGG pathways on CLL dataset

	100%	50%		70%	
	Ground Truth	Observed	Imputed	Observed	Imputed
33	TNF signaling pathway_Homo sapiens_hsa04668				
Overlap	12/110	12/110	8/110	16/110	15/110
Adj. p-value	1.13E-04	2.81E-04	2.25E-02	1.94E-07	1.27E-06
Comb. score	11.69	17.22	2.60	28.21	24.78
34	Cytosolic DNA-sensing pathway_Homo sapiens_hsa04623				
Overlap	9/64	6/64	6/64	7/64	7/64
Adj. p-value	1.58E-04	1.99E-02	2.02E-02	2.87E-03	3.04E-03
Comb. score	5.45	0.03	0.22	-5.01	-4.50
35	Endometrial cancer_Homo sapiens_hsa05213				
Overlap	8/52	5/52	8/52	6/52	7/52
Adj. p-value	2.19E-04	3.10E-02	4.52E-04	4.41E-03	9.32E-04
Comb. score	5.51	-1.45	13.31	-7.65	-4.28
36	TGF-beta signaling pathway_Homo sapiens_hsa04350				
Overlap	10/84	11/84	10/84	11/84	10/84
Adj. p-value	2.37E-04	1.27E-04	4.75E-04	4.27E-05	1.81E-04
Comb. score	8.13	18.51	13.58	10.66	4.50
37	p53 signaling pathway_Homo sapiens_hsa04115				
Overlap	9/69	8/69	6/69	9/69	8/69
Adj. p-value	2.67E-04	2.23E-03	2.47E-02	2.22E-04	9.81E-04
Comb. score	5.17	7.30	-0.65	2.78	-2.47
38	Small cell lung cancer_Homo sapiens_hsa05222				
Overlap	10/86	4/86	4/86	8/86	12/86
Adj. p-value	2.76E-04	2.94E-01	3.01E-01	3.51E-03	1.24E-05
Comb. score	7.16	-2.41	-2.36	-2.15	16.50
39	MicroRNAs in cancer_Homo sapiens_hsa05206				
Overlap	20/297	13/297	17/297	20/297	20/297
Adj. p-value	3.03E-04	8.87E-02	7.41E-03	2.54E-04	2.31E-04
Comb. score	9.57	0.32	7.32	7.94	6.54
40	Cell cycle_Homo sapiens_hsa04110				
Overlap	12/124	11/124	9/124	12/124	16/124
Adj. p-value	3.07E-04	2.08E-03	1.72E-02	2.56E-04	1.12E-06
Comb. score	7.19	9.20	3.71	5.49	22.90
41	Toll-like receptor signaling pathway_Homo sapiens_hsa04620				
Overlap	11/106	9/106	6/106	13/106	14/106
Adj. p-value	3.17E-04	6.52E-03	1.14E-01	1.57E-05	3.99E-06
Comb. score	7.80	5.91	-0.90	15.10	20.81
42	Leishmaniasis_Homo sapiens_hsa05140				
Overlap	9/73	7/73	6/73	8/73	9/73
Adj. p-value	3.69E-04	9.35E-03	3.05E-02	1.49E-03	2.92E-04
Comb. score	4.85	2.53	-0.37	0.14	1.11
43	Pertussis_Homo sapiens_hsa05133				
Overlap	9/75	9/75	6/75	11/75	10/75
Adj. p-value	4.46E-04	9.43E-04	3.35E-02	1.57E-05	7.58E-05
Comb. score	3.54	10.33	-1.02	10.79	4.59
44	NF-kappa B signaling pathway_Homo sapiens_hsa04064				
Overlap	10/93	13/93	9/93	11/93	12/93
Adj. p-value	4.65E-04	1.48E-05	3.53E-03	9.93E-05	2.19E-05
Comb. score	4.97	23.47	7.54	7.73	11.86
45	Tuberculosis_Homo sapiens_hsa05152				
Overlap	14/178	14/178	12/178	16/178	15/178
Adj. p-value	6.64E-04	1.34E-03	8.98E-03	5.51E-05	1.73E-04
Comb. score	6.40	11.29	5.87	11.16	6.87
46	Toxoplasmosis_Homo sapiens_hsa05145				
Overlap	11/118	9/118	8/118	14/118	14/118
Adj. p-value	7.44E-04	1.14E-02	3.03E-02	1.15E-05	1.24E-05
Comb. score	5.83	3.97	1.47	17.30	17.67
47	HIF-1 signaling pathway_Homo sapiens_hsa04066				
Overlap	10/103	9/103	8/103	13/103	11/103
Adj. p-value	1.02E-03	6.00E-03	1.82E-02	1.24E-05	1.99E-04
Comb. score	5.01	6.50	3.74	15.51	4.90
48	Chagas disease (American trypanosomiasis)_Homo sapiens_hsa05142				
Overlap	10/104	11/104	8/104	16/104	13/104
Adj. p-value	1.08E-03	6.01E-04	1.85E-02	1.27E-07	1.48E-05
Comb. score	4.54	13.62	3.28	30.48	15.87

	100%	50%		70%	
	Ground Truth	Observed	Imputed	Observed	Imputed
49	Epithelial cell signaling in Helicobacter pylori infection_Homo sapiens_hsa05120				
Overlap	8/68	7/68	5/68	8/68	9/68
Adj. p-value	1.09E-03	6.79E-03	7.11E-02	9.75E-04	1.82E-04
Comb. score	1.45	3.08	-2.43	-0.06	1.77
50	Salmonella infection_Homo sapiens_hsa05132				
Overlap	9/86	8/86	7/86	9/86	11/86
Adj. p-value	1.10E-03	6.52E-03	2.14E-02	9.91E-04	4.67E-05
Comb. score	2.16	3.90	1.37	0.95	6.85
51	Melanoma_Homo sapiens_hsa05218				
Overlap	8/71	6/71	8/71	9/71	9/71
Adj. p-value	1.41E-03	3.02E-02	3.12E-03	2.56E-04	2.38E-04
Comb. score	2.02	1.06	9.51	3.45	1.81
52	PI3K-Akt signaling pathway_Homo sapiens_hsa04151				
Overlap	20/341	20/341	17/341	24/341	22/341
Adj. p-value	1.43E-03	2.71E-03	2.14E-02	3.31E-05	1.99E-04
Comb. score	5.83	10.90	4.25	15.24	7.85
53	Adherens junction_Homo sapiens_hsa04520				
Overlap	8/74	6/74	6/74	4/74	3/74
Adj. p-value	1.73E-03	3.36E-02	3.19E-02	1.81E-01	4.20E-01
Comb. score	0.42	-0.06	-0.67	-4.33	-2.99
54	Longevity regulating pathway - mammal_Homo sapiens_hsa04211				
Overlap	9/94	7/94	8/94	11/94	9/94
Adj. p-value	1.88E-03	3.10E-02	1.20E-02	1.05E-04	1.70E-03
Comb. score	2.20	1.79	5.09	8.44	0.68
55	Natural killer cell mediated cytotoxicity_Homo sapiens_hsa04650				
Overlap	11/135	3/135	5/135	6/135	7/135
Adj. p-value	1.89E-03	8.45E-01	3.93E-01	1.92E-01	9.30E-02
Comb. score	2.96	-0.72	-1.57	-2.36	-2.56
56	Ras signaling pathway_Homo sapiens_hsa04014				
Overlap	15/227	12/227	15/227	16/227	14/227
Adj. p-value	1.96E-03	3.82E-02	3.99E-03	7.12E-04	4.73E-03
Comb. score	3.75	2.36	9.48	5.87	1.42
57	Jak-STAT signaling pathway_Homo sapiens_hsa04630				
Overlap	12/158	7/158	7/158	10/158	11/158
Adj. p-value	1.97E-03	2.03E-01	2.02E-01	1.49E-02	5.35E-03
Comb. score	3.17	-0.82	-0.99	-0.39	0.30
58	Thyroid hormone signaling pathway_Homo sapiens_hsa04919				
Overlap	10/118	10/118	7/118	10/118	10/118
Adj. p-value	2.29E-03	4.36E-03	7.34E-02	2.19E-03	2.22E-03
Comb. score	1.61	7.39	-0.25	1.23	1.04
59	Apoptosis_Homo sapiens_hsa04210				
Overlap	11/140	13/140	10/140	18/140	15/140
Adj. p-value	2.36E-03	5.83E-04	1.32E-02	1.94E-07	1.71E-05
Comb. score	2.62	15.57	5.80	29.92	17.09
60	Non-alcoholic fatty liver disease (NAFLD)_Homo sapiens_hsa04932				
Overlap	11/151	11/151	7/151	16/151	13/151
Adj. p-value	4.18E-03	6.78E-03	1.76E-01	1.06E-05	3.81E-04
Comb. score	1.85	6.23	-0.83	19.29	5.17
61	Hippo signaling pathway_Homo sapiens_hsa04390				
Overlap	11/153	13/153	14/153	12/153	13/153
Adj. p-value	4.57E-03	1.10E-03	3.99E-04	1.49E-03	4.28E-04
Comb. score	0.95	11.26	14.77	2.61	3.71
62	Focal adhesion_Homo sapiens_hsa04510				
Overlap	13/202	6/202	9/202	12/202	9/202
Adj. p-value	4.86E-03	5.75E-01	1.49E-01	1.17E-02	1.15E-01
Comb. score	1.50	-1.12	-0.51	-0.21	-2.34
63	FoxO signaling pathway_Homo sapiens_hsa04068				
Overlap	10/133	8/133	9/133	11/133	14/133
Adj. p-value	5.12E-03	5.27E-02	2.25E-02	1.56E-03	3.59E-05
Comb. score	0.61	0.90	2.82	2.28	12.44
64	Tight junction_Homo sapiens_hsa04530				
Overlap	10/139	6/139	5/139	8/139	9/139
Adj. p-value	6.88E-03	2.54E-01	4.09E-01	4.54E-02	1.81E-02
Comb. score	0.18	-1.22	-1.56	-2.11	-1.68
65	Phospholipase D signaling pathway_Homo sapiens_hsa04072				
Overlap	10/144	4/144	7/144	7/144	8/144
Adj. p-value	8.76E-03	6.70E-01	1.50E-01	1.16E-01	5.33E-02
Comb. score	0.50	-1.06	-0.78	-2.41	-2.32

Table-T7: KEGG pathways on CLL dataset (continued from Table-T6)

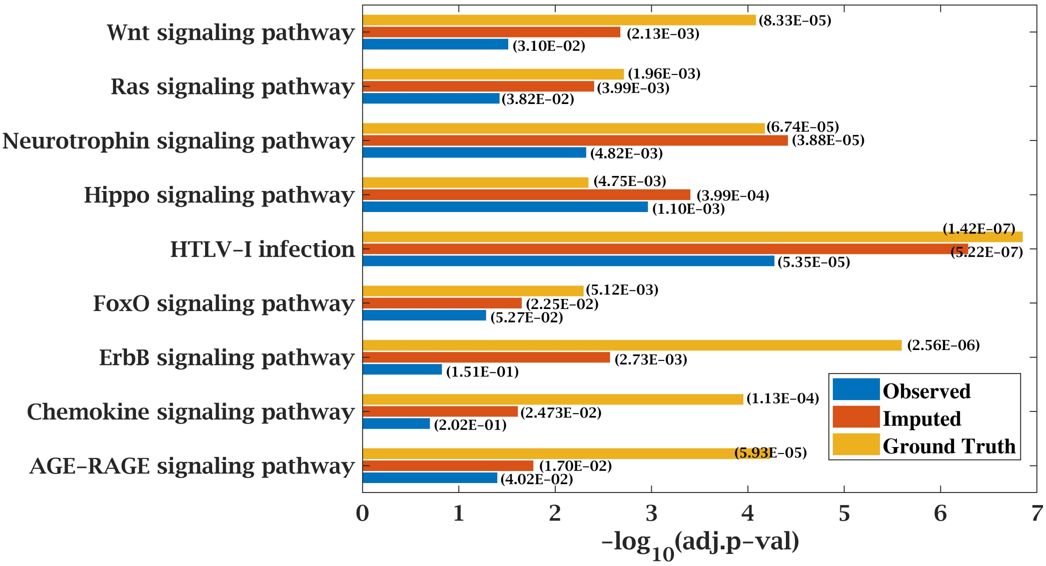

Figure S1. Few important KEGG pathways at 50% observed and imputed data for CLL data. Adjusted p-values are shown in brackets.

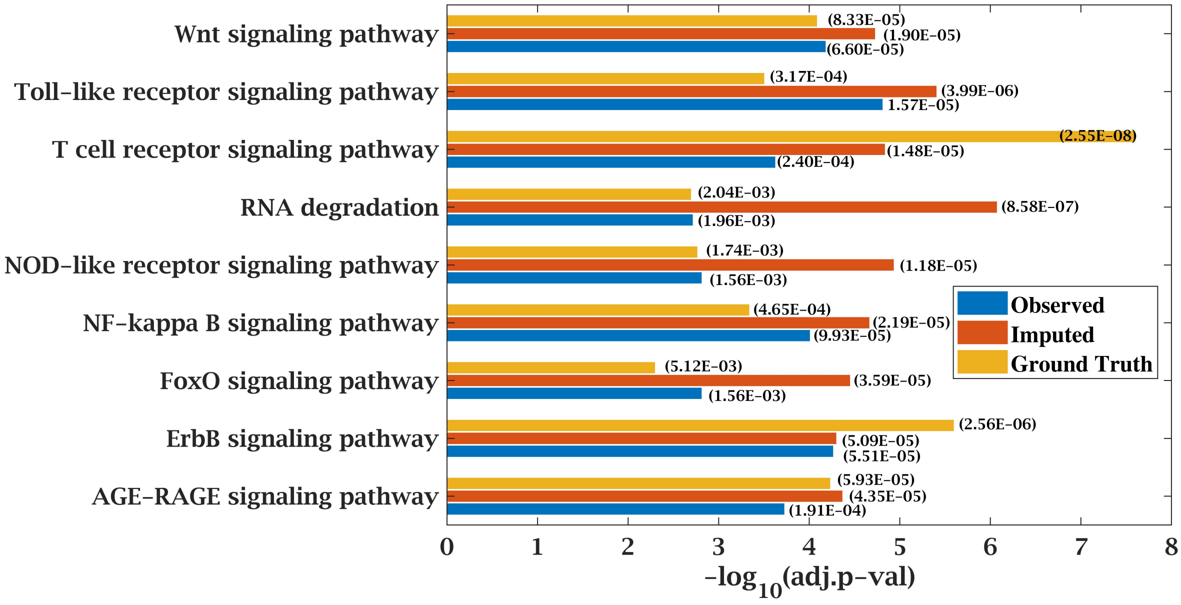

Figure S2. Few important KEGG pathways at 70% observed and imputed data for CLL data. Adjusted p-values are shown in brackets.

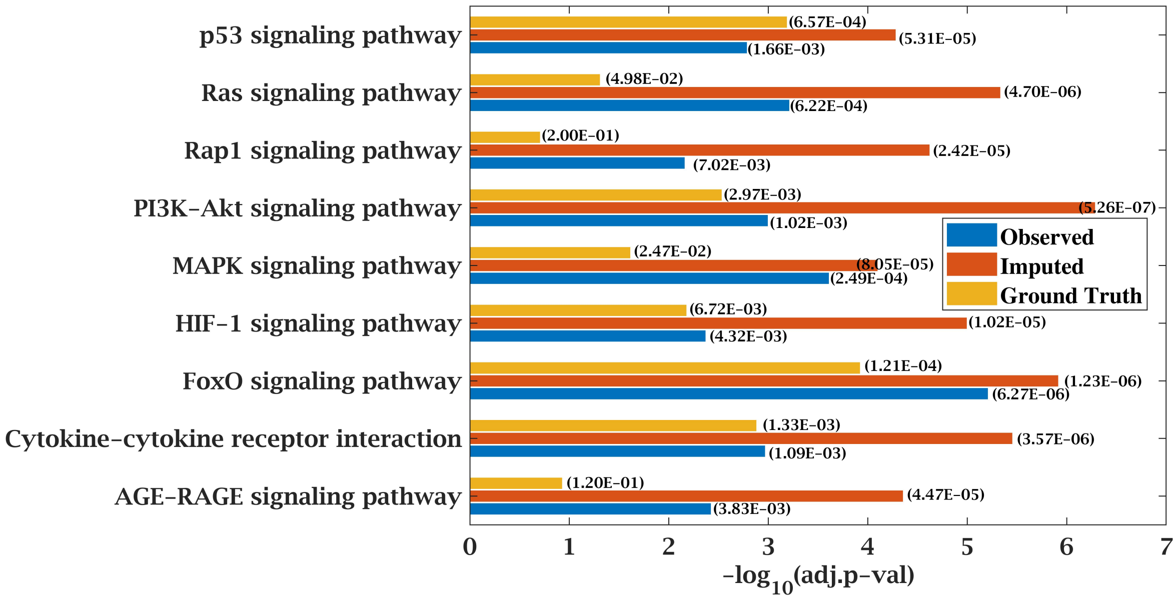

Figure S3. Few important KEGG pathways at 70% observed and imputed data for AML data. Adjusted p-values are shown in brackets.

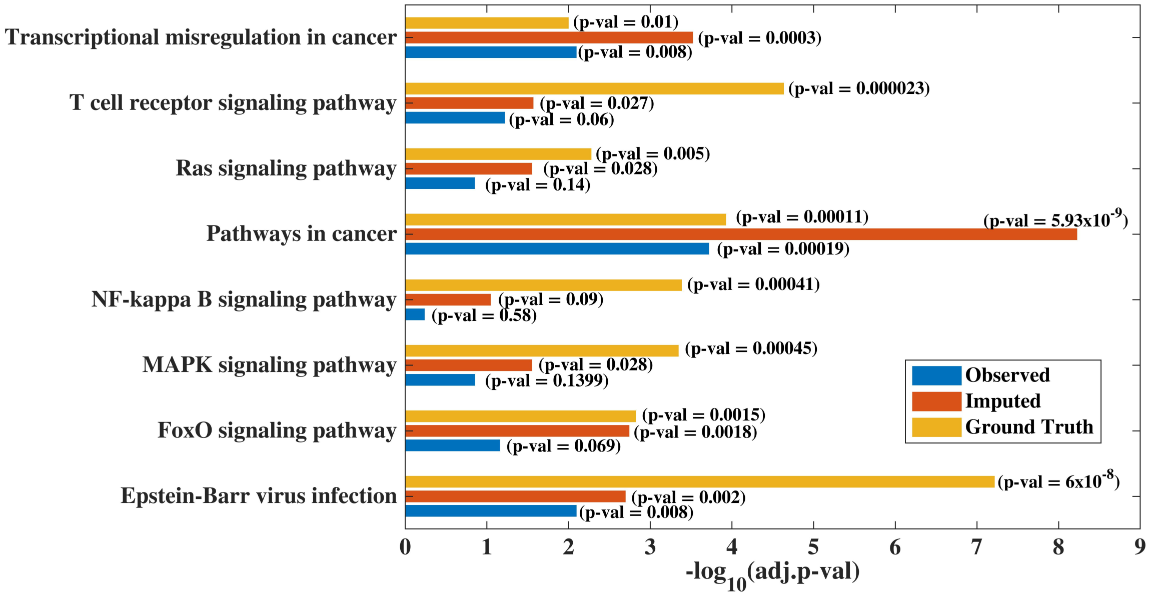

Figure S4. Few important KEGG pathways at 50% observed and imputed data for MM- Spanish data. Adjusted p-values are shown in brackets.

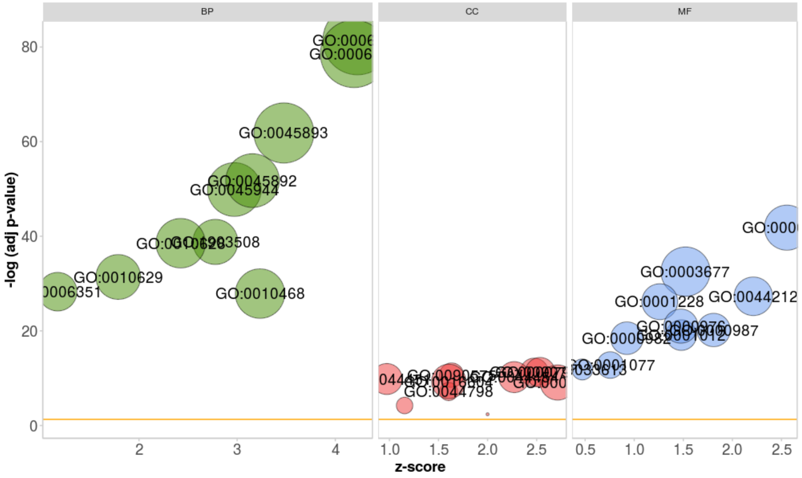

(a) Original

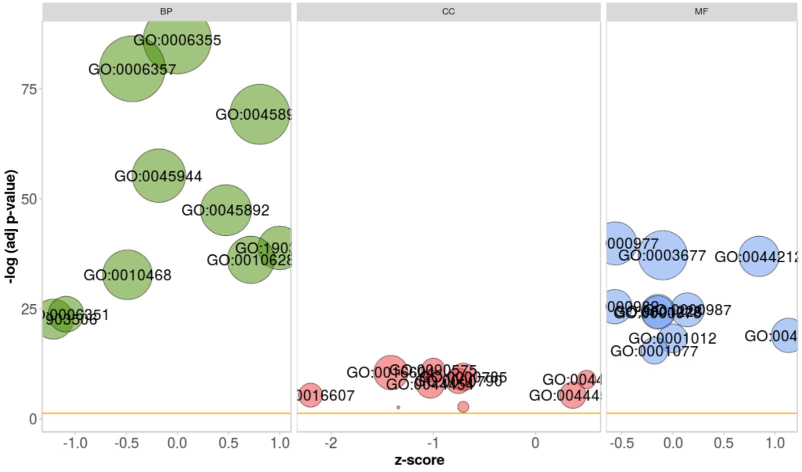

(b) 50% observed input

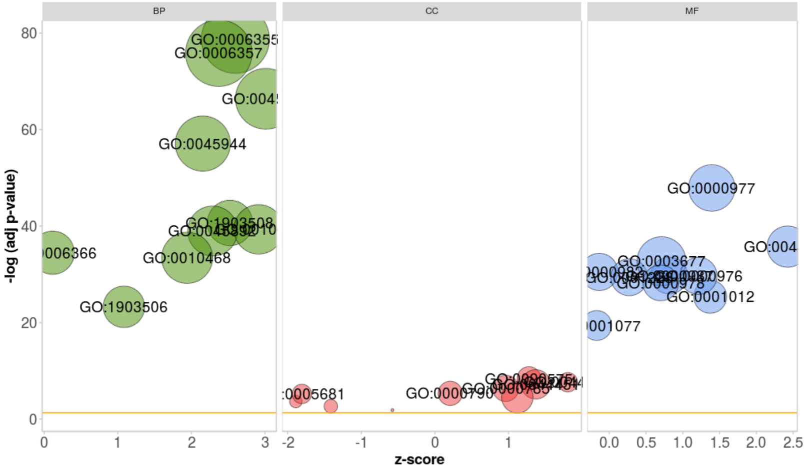

(c)Imputed data

Figure S5. GO bubble plots on CLL dataset. Only top 50 significant pathways are used to draw this bubble plot. Green Bubbles (BP-Biological process), Red bubbles (CC-Cellular Component) and Blue bubbles (MF- Molecular Function)

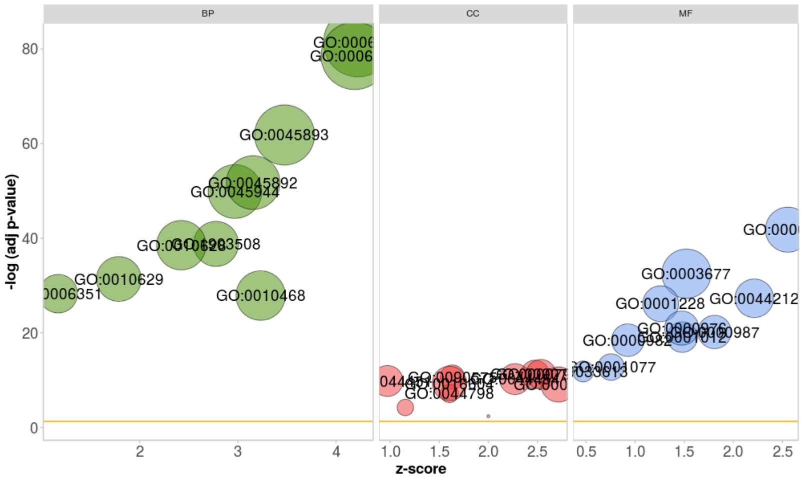

(a) Original

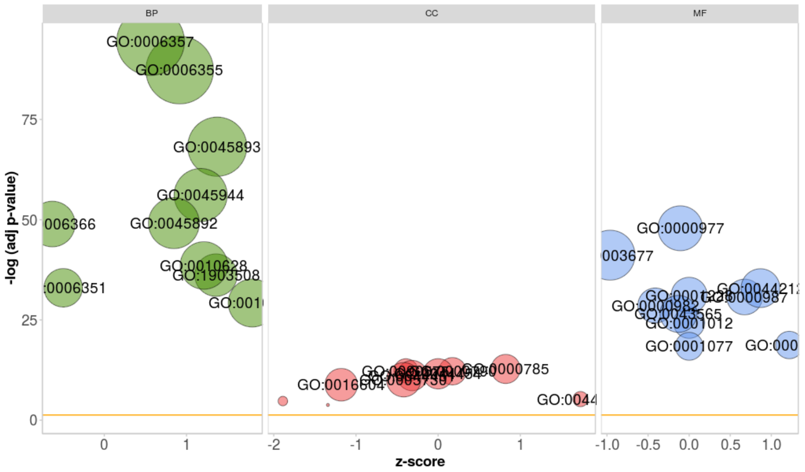

(b) 70% observed input

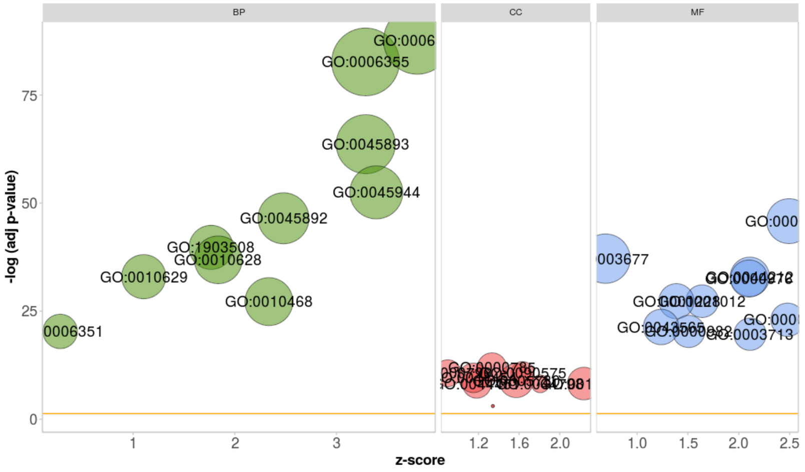

(c)Imputed data

Figure S6. GO bubble plots on CLL dataset. Only top 50 significant pathways are used to draw this bubble plot. Green Bubbles (BP-Biological process), Red bubbles (CC-Cellular Component) and Blue bubbles (MF- Molecular Function)

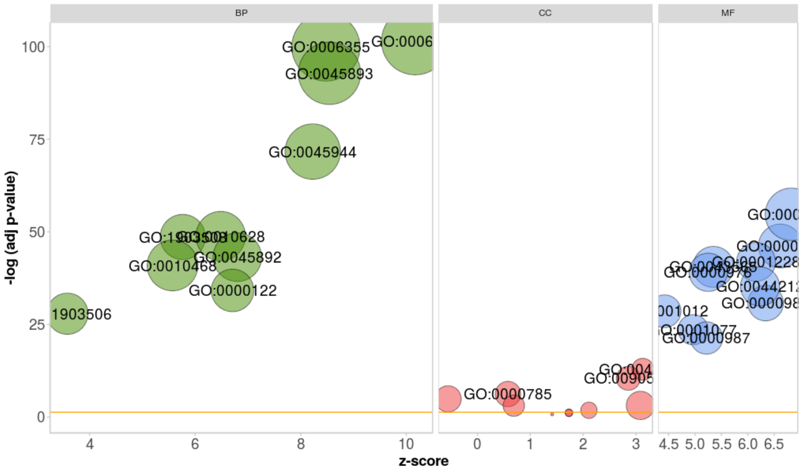

(a) Original

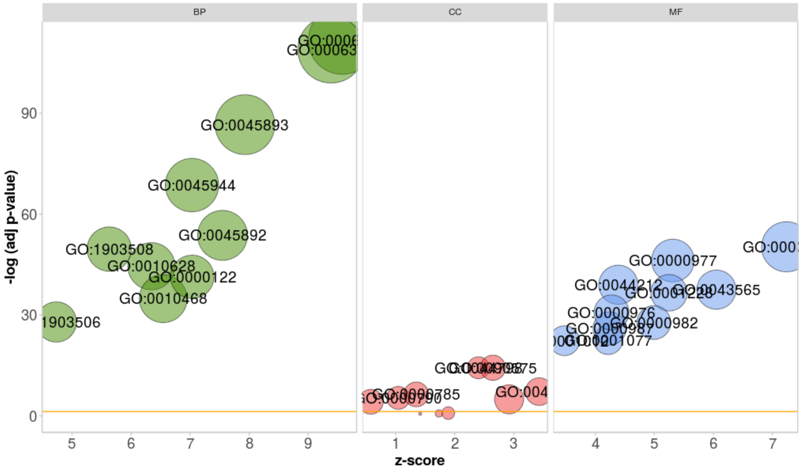

(b) 50% observed input

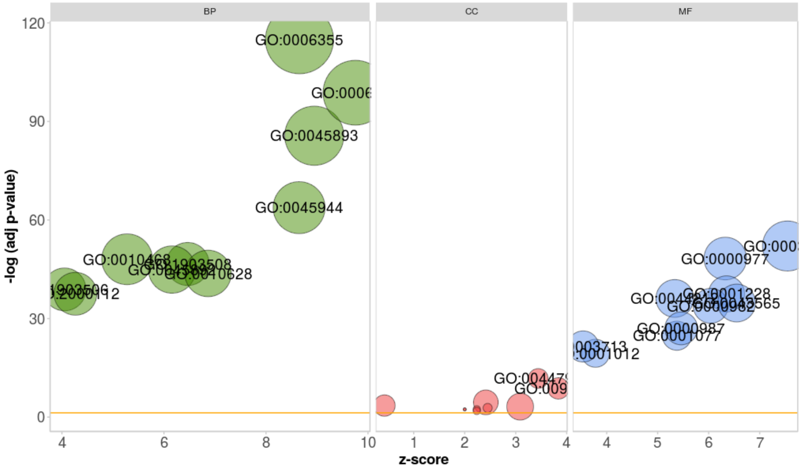

(c)Imputed data

Figure S7. GO bubble plots on AML dataset. Only top 50 significant pathways are used to draw this bubble plot. Green Bubbles (BP-Biological process), Red bubbles (CC-Cellular Component) and Blue bubbles (MF- Molecular Function)

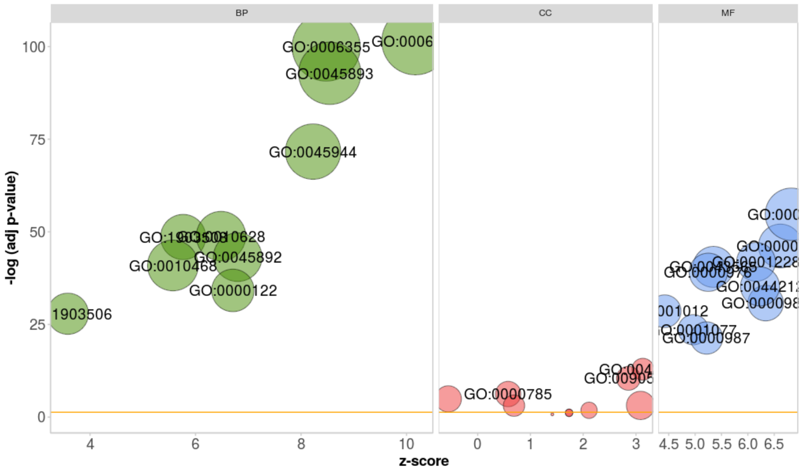

(a) Original

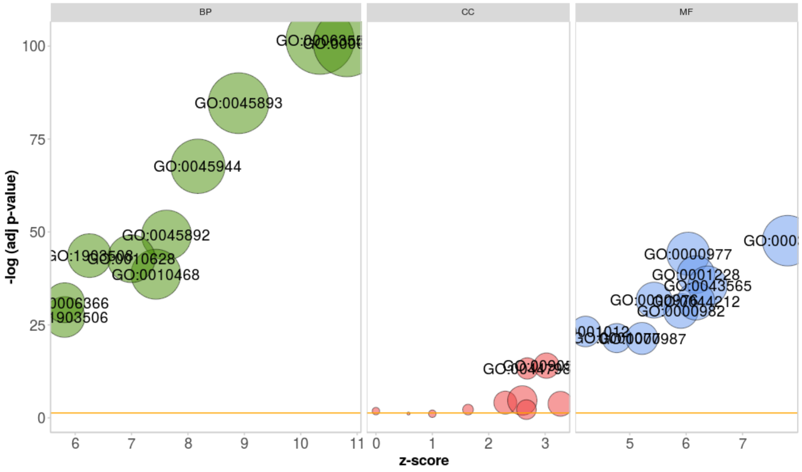

(b) 70% observed input

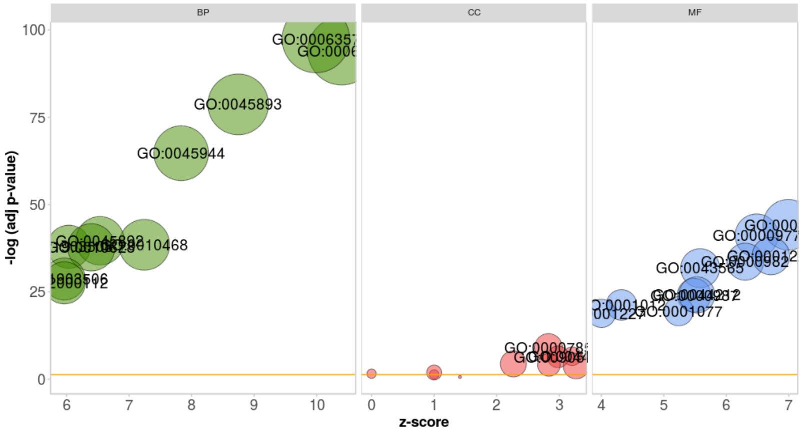

(c)Imputed data

Figure S8. GO bubble plots on AML dataset. Only top 50 significant pathways are used to draw this bubble plot. Green Bubbles (BP-Biological process), Red bubbles (CC-Cellular Component) and Blue bubbles (MF- Molecular Function)

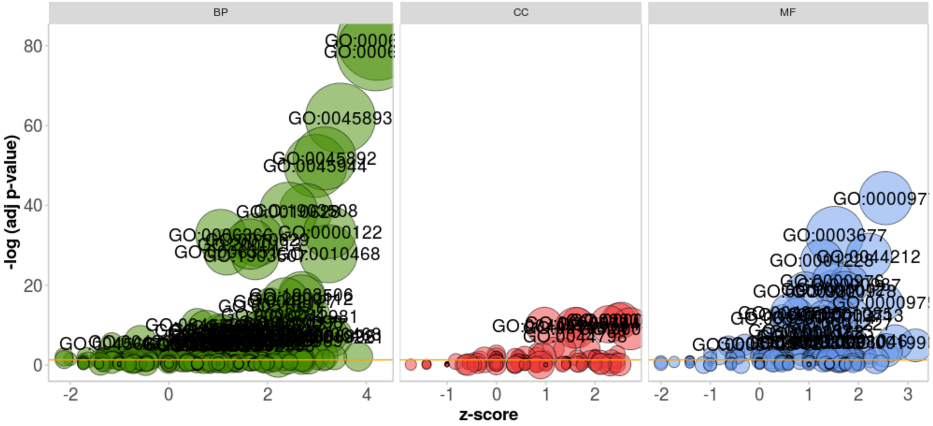

(a) Original

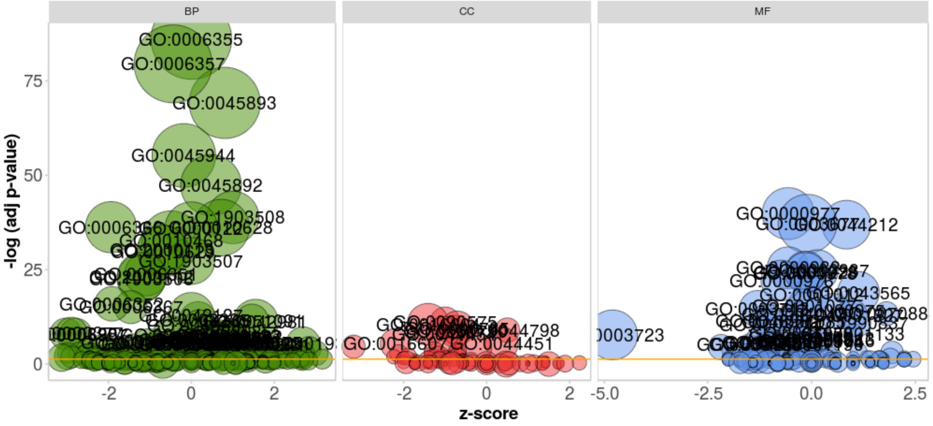

(b) 50% observed input

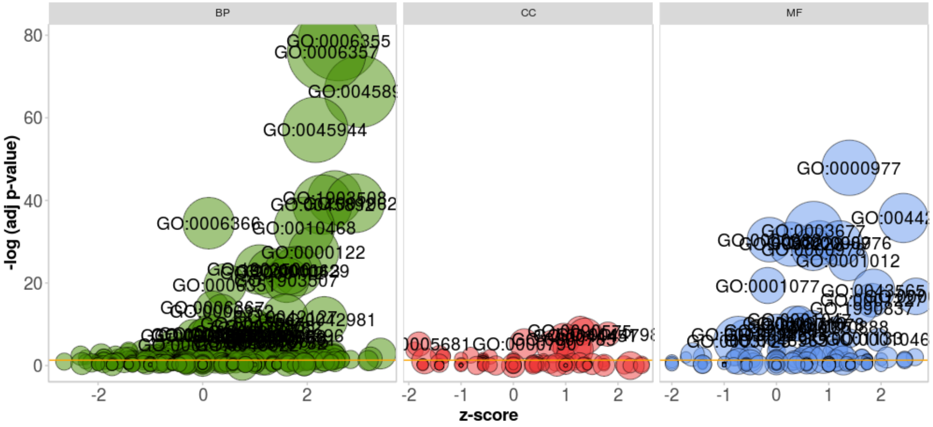

(c)Imputed data

Figure S9. GO bubble plots on CLL dataset. All significant pathways are used to draw this bubble plot. Green Bubbles (BP-Biological process), Red bubbles (CC-Cellular Component) and Blue bubbles (MF-Molecular Function)

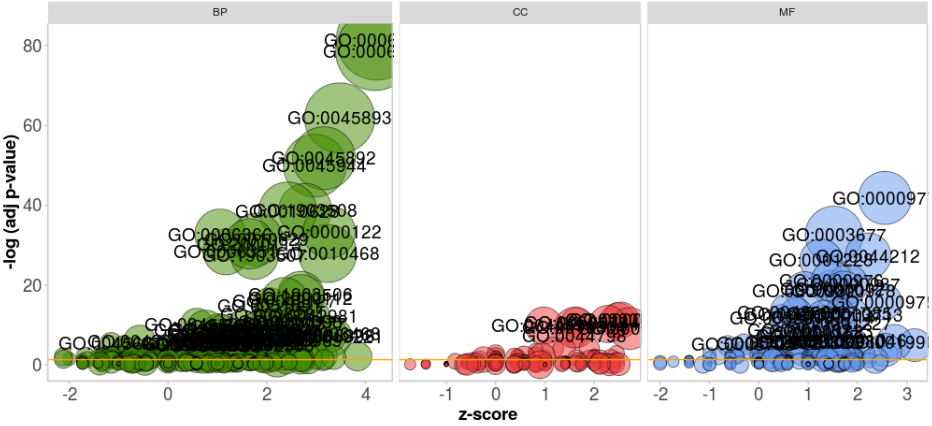

(a) Original

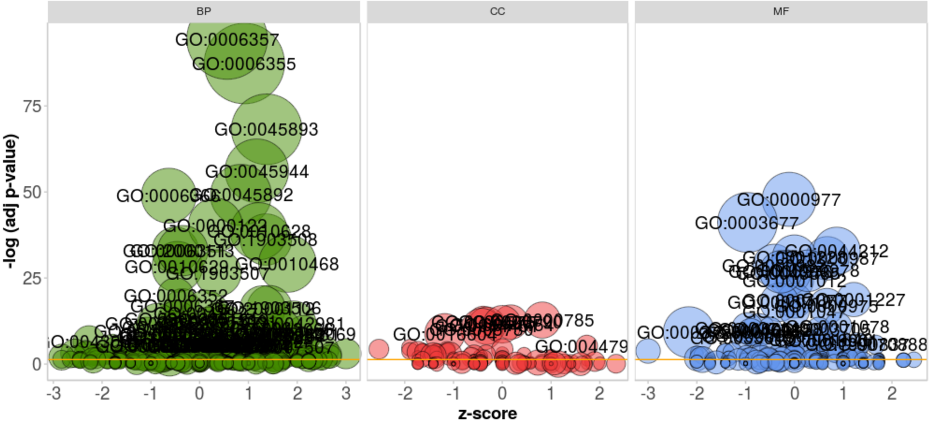

(b) 70% observed input

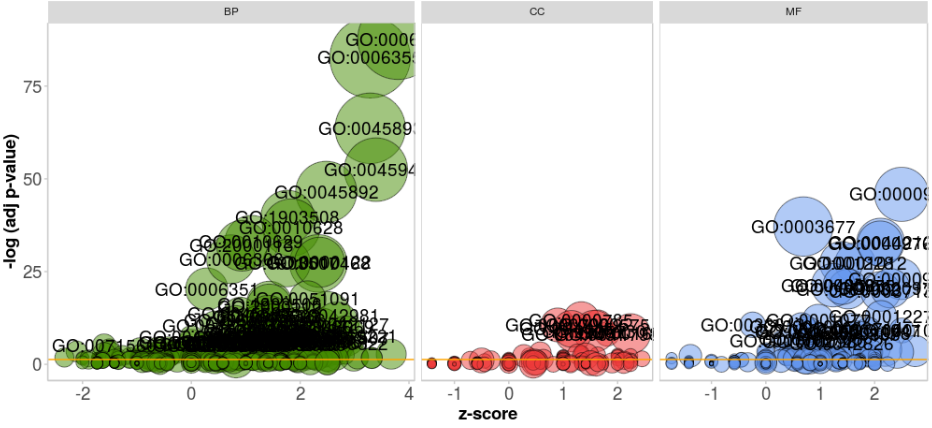

(c)Imputed data

Figure S10. GO bubble plots on CLL dataset. All significant pathways are used to draw this bubble plot. Green Bubbles (BP-Biological process), Red bubbles (CC-Cellular Component) and Blue bubbles (MF-Molecular Function)

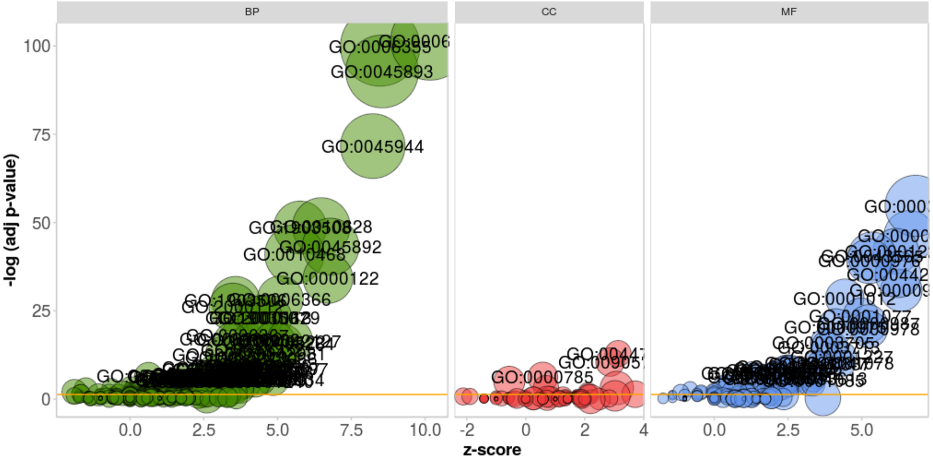

(a) Original

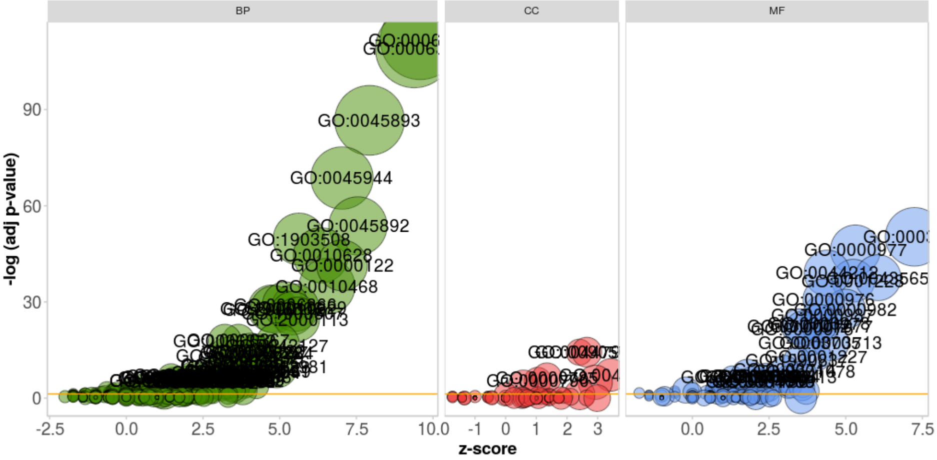

(b) 50% observed input

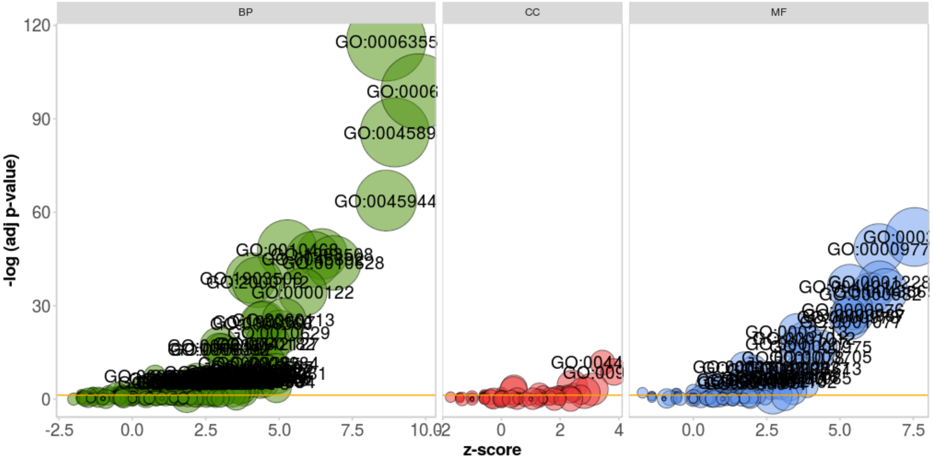

(c)Imputed data

Figure S11. GO bubble plots on AML dataset. All significant pathways are used to draw this bubble plot. Green Bubbles (BP-Biological process), Red bubbles (CC-Cellular Component) and Blue bubbles (MF-Molecular Function)

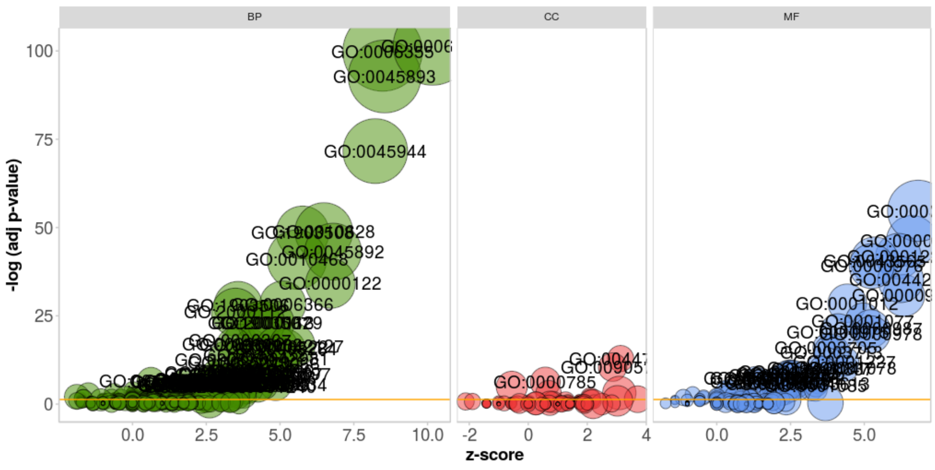

(a) Original

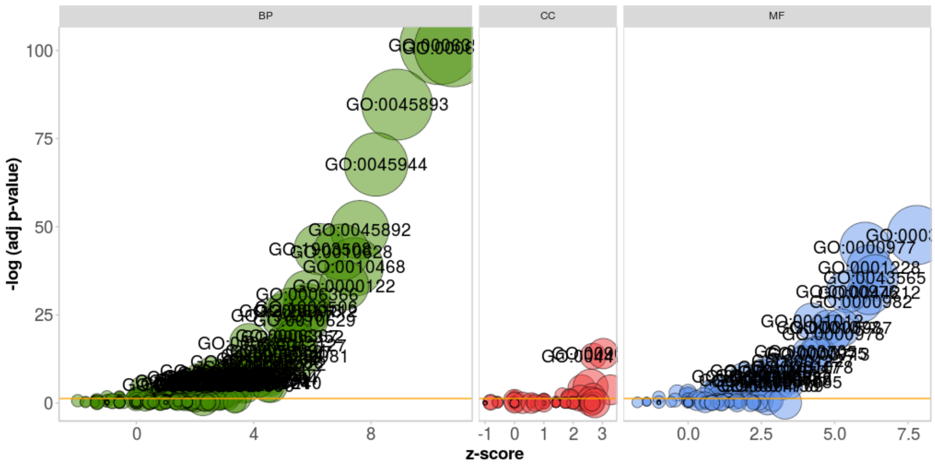

(b) 70% observed input

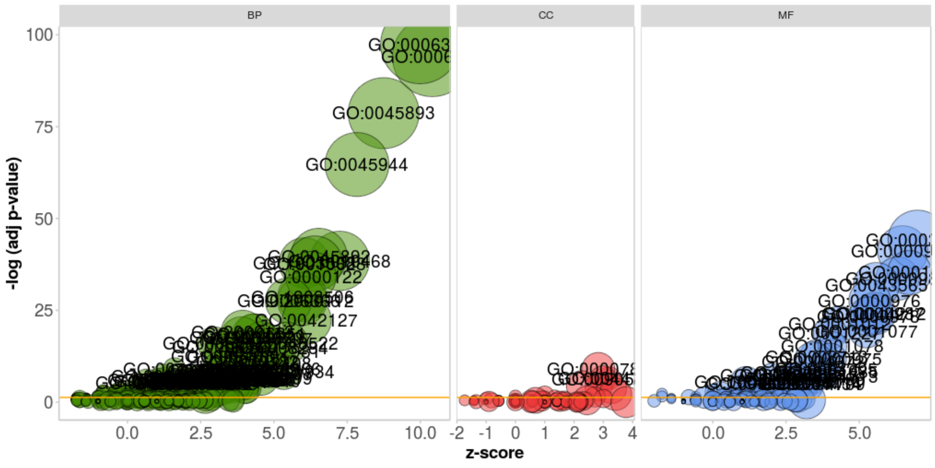

(c)Imputed data

Figure S12. GO bubble plots on AML dataset. All significant pathways are used to draw this bubble plot. Green Bubbles (BP-Biological process), Red bubbles (CC-Cellular Component) and Blue bubbles (MF-Molecular Function)
